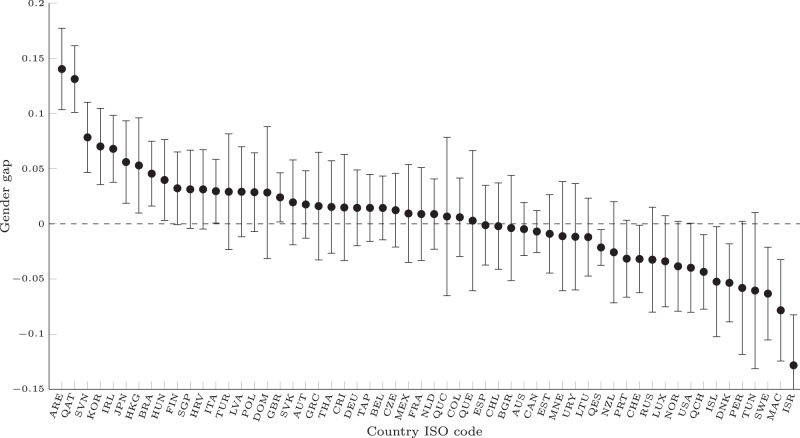# Addendum: Females show more sustained performance during test-taking than males

**DOI:** 10.1038/s41467-021-26458-7

**Published:** 2022-01-18

**Authors:** Pau Balart, Matthijs Oosterveen

**Affiliations:** 1 Departament d’Economia de l’Empresa, Universitat de les Illes Balears, Ctra de Valldemossa km 7,5, 07122 Palma, Spain; 2 Department of Economics, Erasmus School of Economics, Erasmus University Rotterdam, Burgemeester Oudlaan 50, Rotterdam, 3062PA Netherlands

**Keywords:** Education, Human behaviour

Addendum to: *Nature Communications* 10.1038/s41467-019-11691-y, published online 3 September 2019.

We would like to make readers aware that after the publication of this Article, there was an update in PISA’s 2018 Technical report to the response time data used for the PISA 2015 and 2018 datasets (https://www.oecd.org/pisa/data/pisa2018technicalreport/PISA2018-TechReport-Annex-K.pdf). The response time variable was found to represent the time spent on the last visit to a test item, rather than the total time spent on the item, as originally described by PISA and as we thought was used in the original version of this Article.

PISA now provides data on the total time spent on an item, and we have demonstrated that the pattern of results shown in Figure 4a in the Original Article does not change when using the time variable of total time spent on the item (Fig. 1).

**Figure 1:**