# Supplementary material for: Females show more sustained performance during test-taking than males
Source: Nat Commun. 2019 Sep 3;10:3798. doi: 10.1038/s41467-019-11691-y (PMC6722057; doi:10.1038/s41467-019-11691-y)
Supplement: Supplementary file 1 — Supplementary Information [file 41467_2019_11691_MOESM1_ESM.pdf]

Supplementary Information

for

Females show more sustained performance during test-taking than males

Balart and Oosterveen

Supplementary Note I: Study 1 for the Other PISA Waves

1. PISA 2009 (Cluster Level). Figure 2 in the main text displays the coefficients of Equation (2) while estimating it at the item level. In this section we estimate it at the cluster level. By doing so, the unit of analysis exactly matches the unit of randomization: y_{hij} measures the number of correct responses divided by the number of questions within a cluster and Q_{ij} represents the position of the cluster within the test $(0, \frac{1}{3}, \frac{2}{3}, 1)$. Supplementary Figure 1 shows an identical pattern to our baseline results. For 70 out of the 74 participating countries, we found that females were better able to sustain their performance. By estimating Equation (2) at the cluster level we lost some power; the gender difference in sustaining ability was statistically significant at the 5% level for 43 countries. This is mostly the consequence of a reduction in the number of observations and variation in Q_{ij}.

2. PISA 2006 and 2012. The results in the main text are based on the PISA 2009. This section continues by applying a similar analysis to the PISA 2006 and 2012.[1] Our purpose is twofold. First, we can test whether our results are robust to the use of different PISA waves. Second, the PISA 2006 focused on science and the PISA 2012 on math, which assures that the distribution of science and non-science questions for the PISA 2006 and math and non-math questions for the PISA 2012 is quite balanced. This feature allows to study the gender differences for science (2006) and math (2012) separately.

Supplementary Figure 2 shows the estimates for the complete test, estimated with Equation (2). It indicates that our previous results are present across the different PISA waves. For all three waves, we found that for more than 96 percent of the countries females were better able to sustain their performance than males, the difference being statistically significant for more than 75 percent of the countries (at the 5% level). There is not a single country where the gender difference in sustaining ability significantly favored males. We can also see that gender differences are quite stable over time. The correlation of β_3 across the three PISA waves is around 0.45.

Supplementary Figure 3 separates the analysis per topic by means of Equation (3). The upper panel displays the results for science (the PISA 2006) and the lower panel displays the estimates for math (the PISA 2012).[2] We see that in both topics males performed better at the beginning of the test, while females were better able to sustain their performance during

[1] For the PISA 2012 the codebooks do not contain information on the ordering of the questions. The OECD provided them to us.

[2] For the PISA 2006 we combined the math and reading questions into one non-science dummy and for the PISA 2012 we combined the science and reading questions into one non-math dummy. We did not show these estimates, but they can easily be accessed in Supplementary Database 1.

the test. In science (math) females were better able to sustain performance during the test in 52 (59) out of 57 (68) countries, being statistically significant for 30 (26) of them at the 5% level. In contrast, there is no country where males exhibited an ability to better sustain their performance for any specific domain in which they performed better on average (statistical significance at the 5% level). This confirms that, separately for math and science, the gender gap at the beginning of the test favors males, but this advantage is either smaller, offset, or reversed at the end of the test.

3. PISA 2015. We complement our analysis by using the most recent PISA wave (2015). For 58 out of 73 participating countries this test was administered on computers. In the main text, we used this computer-based wave to investigate potential determinants of the gender difference. Together with the implementation of the computer-based test, the PISA introduced a few other changes to the test design.[3] All the characteristics necessary to implement Study 1 remained present: clusters of questions vary between the booklets and booklets are randomly assigned to students. Supplementary Figure 4 and 5 show that our baseline results from the previous waves carry over to the PISA 2015. The smaller number of countries for which the gender difference is present when considering science questions only (Supplementary Figure 5) is partly explained by the sample of countries that administered the computer-based test and partly by an increase in the estimated standard errors.

Supplementary Note II: Potential Determinants of the Gender Difference in Study 1

In this section, we provide further detail on the three potential determinants of the gender differences in ability to sustain performance. These determinants were discussed at the end of the Results section of Study 1 in the main text.

1. Noncognitive Skills. To test whether noncognitive skills could explain the gender difference documented in Study 1, we estimated Equation (2) of the main text while separately including the individual measures of noncognitive skills (NC) and their interaction with the position of

[3]Three changes were implemented. First, next to science, math, and reading the PISA 2015 introduced a new domain called collaborative problem solving. As not all countries participated with this new domain and it was not represented in previous waves, we only included in our analysis the booklets that do not contain the clusters related to collaborative problem solving. Second, the PISA used 35 different booklets per country, which is substantially more than the 13 booklets used in the previous waves. Third, a somewhat more sophisticated rotation design made it possible for a cluster of questions to be at the same position in more than one booklet. A student was randomly assigned to one of the 35 booklets, this determined the position of the science clusters and the position and exact id of the math and reading clusters. A second random number for the student combined with his or her booklet number determined the exact id of the science clusters. See the PISA 2015 technical report for more details [1].

the question: $y_{hij} = \beta_0 + \beta_1 F_i + \beta_2 Q_{ij} + \beta_3 Q_{ij} F_i + \beta_4 \text{NC}_i + \beta_5 Q_{ij} \text{NC}_i + \epsilon_{hij}$. An insignificant estimate for β_3 across countries would indicate that the corresponding measures of noncognitive skills were mediating the gender difference in ability to sustain performance during the test. The country estimates for all the analyses reported below are available in Supplementary Database 2.

1.1. Validated Measures (see Supplementary Table 6 for an overview). As explained in the main text, one major advantage of the PISA is that the student background questionnaires were used to construct validated measures of several noncognitive skills. The available constructs vary across the PISA waves. To provide us with a broad range of noncognitive skills, we extracted these validated measures for the PISA waves 2006, 2009, and 2012. The technical report of each PISA wave describes in detail the construction of the measures and the validation exercises (Chapter 16: Scaling Procedures and Construct Validation of Context Questionnaire data [2, 3, 4]). In the main text these measures were shortly introduced via bullet points. Here they are discussed in more detail in the same order as in the main text. Supplementary Table 6 provides an overview of all the measures and their underlying items. Supplementary Table 6 also documents for each measure the average gender difference across all PISA countries. For most of the noncognitive skills we find gender differences that are consistent with the previous literature, which implies our data confirms that they are possible candidates to mediate the findings of Study 1. See the main text for further discussion on this.

We started out by testing the importance of favorable attitudes towards education with three different measures. First, the PISA 2006, 2009, and 2012 measured students' interest towards the topics science, reading, and math respectively. For instance, the student questionnaire of the PISA 2006 contained the item "*I have interest in the way scientists do experiments*", the PISA 2009 contained "*For me, reading is a waste of time*", and the PISA 2012 included "*I do mathematics because I enjoy it*". Using the answers to these questions together with several other items, the PISA provides validated measures of a student's interest in science, reading, and math. Second, the PISA 2006 and 2012 contained measures of instrumental motivation towards math and science, for example one item was "*I will learn many things in my science/math courses that will help me get a job*". Third, the PISA 2009 and 2012 measured general attitudes towards school and learning. One item used to construct the former measure was "*School has been a waste of time*", where an item used in the latter was "*I enjoy receiving good grades*". Our findings indicated, however, that none of these measures can mediate the gender difference in ability to sustain performance (the estimates for all the analyses with noncognitive skills are available in Supplementary Database 2). For instance, female students reported a higher interest in reading, and students with a higher interest in reading were also better able to

sustain their performance during the test in 42 countries (statistically significant at the 5% level). However, we found that after controlling for this, the baseline gender difference was still present and statistically significant at the 5% level in 47 countries.

Next, we used measures of self-efficacy and self-concept. The two capture students' beliefs about their own ability in a specific domain. The main difference is that self-concept is focused on the beliefs about ones' general ability in that domain, where self-efficacy refers to beliefs in a specific context [5]. As a consequence, the items for self-efficacy are task specific. The PISA 2012 measured self-efficacy and self-concept in the domain of mathematics. One item used to construct the measure for self-efficacy was "*Are you confident to calculate how much cheaper a TV would be after a 30% discount*" while one item used for self-concept was "*I have always believed that mathematics is one of my best subjects*". Our estimates in Supplementary Database 2 showed that self-efficacy and self-concept in mathematics could not explain the gender difference.

Next, we considered constructs that measure a student's intention towards their future studies and jobs. The student questionnaire of the PISA 2006 contained a measure for future career intentions in science, where one item was "*I would like to study science after secondary school*". The PISA 2012 contained a construct about students' future career intentions in general. One of the questions included to construct the measure was "*I spoke to a career adviser at my school*". Our results indicated that favorable intentions towards future studies and jobs cannot parse out the gender difference.

Finally, the PISA 2012 contains measures for four well-known noncognitive skills: conscientiousness, openness, neuroticism and locus of control. The student questionnaire of the PISA 2012 contained five items to measure conscientiousness, one of them was: "*When confronted with a problem, I give up easily*". The construct for openness was focused on the domain of problem solving and contained, among others, the question "*I can easily link facts together*". Both neuroticism and locus of control were specifically focused in the domain of mathematics. One of the items used to construct the measure for neuroticism was "*I get very nervous doing mathematics problems*" and one item used for locus of control was "*I can perform bad on a mathematics quiz, because sometimes I am just unlucky*". Our results indicated that none of these well-known noncognitive skills could mediate the gender difference. Supplementary Table 6 documents that the measures for conscientiousness, openness, and internal locus of control favored males in our data. For openness and locus of control this might be explained by their focus on the domain of problem solving and mathematics respectively. We describe alternative measures for the three noncognitive skills below.

1.2. Separate Items as Measures (see Supplementary Table 7 for an overview). Where none of the validated constructs above were able to explain the gender differences in the ability to sustain performance during the test, one might argue that two relevant skills of the Big Five taxonomy were not controlled for: agreeableness and extraversion. To address this, at least partially, we drew upon individual items as proxies for these two noncognitive skills. The student questionnaire of the PISA 2009 contained the item "*I get along well with most of my teachers*", which we used for agreeableness, and the PISA 2012 included "*I make friends easily at school*", which was used for extraversion. As recognized in the main text, using a single item limits the scope of this part of the analysis. However, for these two skills, single items constitute the only available measure. This imposes some caution in interpreting the results of this analysis.

Moreover, we drew upon individual items for openness and locus of control, as the validated measures above for these two noncognitive skills focused, respectively, on the domain of problem solving and mathematics. For openness we had three items in the PISA 2009, which included for instance "*I learn about things that are not course-related, such as sports, hobbies, people or music*". Locus of control was measured by six items in the PISA 2012, such as "*It is completely my choice whether or not I do well at school*". As we had multiple items for openness and locus of control, we also constructed the first principal component across items for these two skills to alleviate concerns related to measurement error (both using regular pca and polychoric pca, where the latter is more appropriate with discrete variables).

Supplementary Table 7 provides an overview of all these individual items, which also demonstrates that they are similar to items in validated scales, such as the Big Five Inventory [6]. The final column of this table documents that for these items females report higher levels of agreeableness, openness (on one of the three items), and internal locus of control. Similarly, as before, the results showed that these measures cannot mediate the gender difference in Study 1. The country estimates are reported in Supplementary Database 2.

1.3. Non-Self-Reported Measure. All previous measures were based on self-reports. Recent research proposes and validates a non-self-reported measure for conscientiousness: careless answering behavior in survey [7, 8, 9]. Following this research, we calculated the proportion of questions that the student did not provide an answer to in the student background questionnaire to construct a non-self-reported measure of conscientiousness. Our data indicate that females show higher levels of conscientiousness on this measure; the proportion of questions that the student did not provide an answer to was roughly 0.9 percentage point lower for females (p-value=0.00, two-sided t-test). As before, we found that this measure was unable to explain the gender difference, further corroborating the findings above. The country estimates are reported

in Supplementary Database 2.

2. Test Taking Strategies. In this subsection we further elaborate upon test taking strategies as a potential determinant for the gender difference. In the main text we defined test taking strategies as any reason that leads a student to answer the questions in a different order than the order proposed by the test. To investigate this potential explanation, we took advantage of the fact that on the computer-based test in the PISA 2015, students were not allowed to go back and forth among units of questions [1]. We should note that questions on the PISA tests are organized into units. Reading units contain 3.5 questions on average while math and science units contain on average 1.6 questions. We estimated Equation (2) for the PISA 2015 at the unit level, where y_{hij} represents the average performance within a unit j, Q_{ij} is the position of the unit within the test, and question fixed effects are replaced by unit fixed effects. To identify the gender difference, we only used the variation in unit ordering across students. As students could not go back and forth between units, we can be sure that the position of the unit in the test is the actual position in which the unit was answered. Supplementary Figure 6 shows an identical pattern to our baseline results, making it implausible that gender differences in test taking strategies significantly drove our results. We did, however, lose significance for seven countries, which is most likely the consequence of a reduction in the number of observations and in the variation in Q_{ij}.

3. Effort During the Test. In this subsection, we provide further detail on how we tested the role of test effort as a potential explanation for the gender difference in sustaining ability during the test. We provide a small theoretical framework to better explain the concepts involved in the analysis.

The computer-based nature of the PISA 2015 allows us to get information on two proxies for effort: time spent per question (T) and actions per question (A). Consider a production function where cognitive skills (C) and the two measures of effort are used as inputs to generate correct answers (Y):

$$Y_Q = \theta_Q \, g(C, T_Q, A_Q)$$

We use the subscript Q to highlight that these variables may change depending on the position of the question in the test (i.e., that they are dynamic). θ_Q is interpreted as a total-factor-productivity parameter, which we view as the efficacy of the mental process that transforms inputs into correct answers. Importantly, it can also vary in Q. This parameter may account for mental fatigue or any other element not fully captured by T_Q and A_Q. Regarding the role of the two dynamic inputs, the technical report of the PISA 2015 [1] has reported that

better performing students generally take more time to complete the test, and in 48 out of 58 countries we found a statistically significant positive correlation between the number of actions and answering a question correctly. These findings indicate that, consistent with the definition of an input, the first partial derivatives of Y_Q with respect to A_Q and T_Q are positive. The derivative of Y_Q with respect to Q can be expressed as:

$$\frac{\partial Y_Q}{\partial Q} = \frac{\partial \theta_Q}{\partial Q} g(C, T_Q, A_Q) + \theta_Q \frac{\partial g(C, T_Q, A_Q)}{\partial T_Q}\frac{\partial T_Q}{\partial Q} + \theta_Q \frac{\partial g(C, T_Q, A_Q)}{\partial A_Q}\frac{\partial A_Q}{\partial Q}$$

One explanation for the gender difference is that females might be better able to keep up their levels of dynamic inputs during the test.[4] This would be the case if $\frac{\partial T_Q}{\partial Q}$ and/or $\frac{\partial A_Q}{\partial Q}$ were greater for females than for males. We tested this possibility by estimating Equation (2), replacing the outcome variable with T_Q and A_Q. The former is measured in minutes, while the latter is a composite measure of the number of clicks, double-clicks, key presses, and drag/drop events.[5]

We found that the number of actions and time spent per question also declined during the test. On average, students used fewer actions and spent less time per question at the end of the test than at the beginning. This finding is consistent with the existence of a decline in performance. However, can the dynamic inputs also explain the gender difference in sustaining ability during the test? Figure 4a in the main text showed that the decline in time spent per question during the test does not follow an obvious gender pattern across countries. Depending on the country, either females or males decreased the amount of time spent per question more quickly, with most of the estimates being statistically insignificant. Figure 4b in the main text revealed that for most countries the number of actions per question during the test dropped faster for females. The pattern was not as strong as our baseline result; we found this in 45 out of 58 countries, being statistically significant for 18 of them at the 5% level.

We conclude that the two dynamic inputs cannot explain the gender difference in sustaining performance during the test. This is confirmed by augmenting Equation (2) with the two measures and estimating $y_{hij} = \beta_0 + \beta_1 F_i + \beta_2 Q_{ij} + \beta_3 Q_{ij} F_i + \beta_4 T_{ij} + \beta_5 Q_{ij} T_{ij} + \beta_6 A_{ij} + \beta_7 Q_{ij} A_{ij} + \epsilon_{hij}$. By doing this, we still found that females were better able to sustain their performance during the test; see Supplementary Figure 7. Then, according to the theoretical framework, we could attribute the gender difference to θ_Q. To make this explicit, we estimated a linear approximation of the relationship between the gender differences in ability to sustain performance and gender differences in sustaining dynamic inputs during the test across countries

[4] By definition, knowledge (C) is constant during the test ($\frac{\partial C}{\partial Q} = 0$).

[5] The PISA interface provides some tools to generate an answer, such as a calculator. This means that the number of actions (A_Q) does not simply mean filling in an item.

(c):

$$\underbrace{\frac{\partial Y_Q}{\partial Q}\Big|_{\text{females}} - \frac{\partial Y_Q}{\partial Q}\Big|_{\text{males}}}_{\widehat{\beta^Y_{3c}}} = \underbrace{\left[\frac{\partial \theta_Q}{\partial Q}\Big|_{\text{females}} - \frac{\partial \theta_Q}{\partial Q}\Big|_{\text{males}}\right]}_{\delta_0} + \underbrace{\left[\frac{\partial T_Q}{\partial Q}\Big|_{\text{females}} - \frac{\partial T_Q}{\partial Q}\Big|_{\text{males}}\right]}_{\delta_1 \widehat{\beta^T_{3c}}} + \underbrace{\left[\frac{\partial A_Q}{\partial Q}\Big|_{\text{females}} - \frac{\partial A_Q}{\partial Q}\Big|_{\text{males}}\right]}_{\delta_2 \widehat{\beta^A_{3c}}} + \epsilon_c$$

The intercept of this OLS regression captures the gender differences in sustaining performance during the test that cannot be explained by the dynamic inputs, but might be related to differences in total factor productivity ($\frac{\partial \theta_Q}{\partial Q}$). A positive intercept is consistent with females being better able to transform inputs into correct answers as the test goes on. Supplementary Figure 8 displays these two regressions visually and clearly shows that the intercept is positive for both dynamic inputs. Also note that, as expected, the gender differences in sustaining performance and in sustaining dynamic inputs during the test show a significant positive relationship.[6] Supplementary Table 8 shows the estimates of the corresponding regressions and confirms the visual results, where columns (1), (3), and (5) display the results for time, number of actions, and both inputs combined. Columns (2), (4), and (6) include an interaction between the gender difference in sustaining dynamic inputs during the test and at the start of the test, which controls for the notion that a drop in inputs might have a larger effect if the starting level of effort is lower.[7] Consistent with diminishing marginal returns to these inputs, we found this interaction was negative: for countries in which females are better able to keep up their inputs during the test, the gender difference in sustaining performance becomes smaller when females also have a higher baseline level of effort. However, the coefficients on the interaction terms are insignificant and do not change the magnitude or significance of the positive intercept.

We view θ_Q as the efficacy of the mental process that translates test inputs into answers. What does this mental process entail? As it cannot be observed in our dataset, we cannot provide a conclusive answer to this question. One possibility is that our finding is related to the literature that has documented a gender difference that arises when considering the temporal dimension in performance, i.e., boredom.

Previous research has found that females experience lower levels of boredom when performing activities with a long duration [10, 11, 12]. Previous works argued that a definition for boredom could be given in terms of attention, as performance on sustained attention tasks (so-called vigilance tasks) associated with common measures of boredom [13].[8] Individuals who experience

[6]Countries for which the decline in the number of actions and time spent per question is stronger for females show a smaller gender difference in ability to sustain performance and vice versa.

[7]This notion of nonlinearity is captured by the conceptual framework as the drop in dynamic inputs ($\frac{\partial T_Q}{\partial Q}$) is multiplied by the change in the production function ($\frac{\partial g(\cdot)}{\partial T_Q}$).

[8]More specifically, the definition has two components: (i) not being able to successfully pay the attention required to participate in a satisfying activity and (ii) being aware of this, resulting in either an attempt to

boredom have impaired performance on various tasks [13, 14, 15]. This literature argues that the response to boredom is different between people who seek external stimulation (agitated boredom) versus internal stimulation (apathetic boredom) [16, 17]. Our results fit well with an agitated type of boredom, where a common response is to force oneself to pay attention to the task at hand [13, 15, 16, 18]. However, our data does not allow us to provide conclusive evidence in favor of this hypothesis.

Supplementary Note III: Robustness of Study 1

This section analyzes the robustness of the gender difference presented in Study 1. In particular, we control for the potential impact of difficult questions, consider nonlinearity in three different ways, analyze unreached questions, use a different definition for the performance at the start of the test, analyze the potential effects of the small break halfway during the PISA test, and investigate potential differences between multiple-choice and open ended questions. Unless noted otherwise, we will use the PISA 2009 throughout this section.

1. Being Stumped. One might consider the possibility that students get demotivated by certain questions on the test, causing them to perform poorly thereafter. If males suffered more greatly from this phenomenon, the gender difference might not be robust to controlling for the impact of such questions.[9] Measuring whether a student got stumped on a question is not easy, but by means of the PISA 2015 we can conceptualize it as the question in which a student put forth more effort (the maximum number of actions) while answering it wrong. We re-estimated Equation (2) while including a dummy S, which equaled 1 after such a question, and interacted it with Q: $y_{hij} = \beta_0 + \beta_1 F_i + \beta_2 Q_{ij} + \beta_3 Q_{ij} F_i + \beta_4 S_{ij} + \beta_5 Q_{ij} S_{ij} + \epsilon_{hij}$. Supplementary Figure 9 documents our estimates for the gender difference are unchanged.

2. Probit. Our main estimates were computed using OLS. As the dependent variable is binary, we also estimated our baseline equations by making use of a probit model. The coefficients for the complete test and per topic are shown in Supplementary Figure 10 and 11 respectively, which are very similar to the ones obtained by OLS. Note that for a probit model the coefficients are not equal to the marginal effects. For the complete test we also tested the significance of the marginal effects by using a Welsch t-test. The results are virtually identical to directly testing the coefficients, see Supplementary Table 11. We provide technical details on the Welsch t-test below.

---

engage with the task at hand or awareness of engagement in matters unrelated to the task.

[9] Previous studies found that females were more likely to stop competing if they lost, suggesting that females would suffer more from being stumped [19].

2.1. Technical Details on the Welsch t-test. To test for differences in marginal effects, we use a similar procedure as described by Norton and co-authors [20]. After estimating the probit model, we took its derivative with respect to Q, which after suppressing subscripts, reads as $\phi(\cdot)(\beta_2 + \beta_3 F)$, where ϕ is the standard normal density function. Subsequently we evaluated this expression for males at $F = 0$, $Q = 0.5$, and $Q * F = 0$ and for females at $F = 1$, $Q = 0.5$, and $Q * F = 0.5$ (for brevity, we skip these arguments and just write "males" or "females" when using this function below). As such, we had a value for the average marginal effect of males ($\frac{1}{N}\sum_{n=1}^{N}\phi(\,\cdot\,|_{\text{males}})\beta_2$) and females ($\frac{1}{N}\sum_{n=1}^{N}\phi(\,\cdot\,|_{\text{females}})(\beta_2+\beta_3)$). In practice, these marginal effects are tested through standard z-tests. Therefore, we performed a simple Welsch t-test on the significant difference of them. More specifically, we applied the Welsch t-test as follows (omitting the summations):

$$\frac{\phi(\,\cdot\,|_{\text{males}})\beta_2 - (\phi(\,\cdot\,|_{\text{females}})\beta_2 + \phi(\,\cdot\,|_{\text{females}})\beta_3)}{\sqrt{Var[\phi(\,\cdot\,|_{\text{males}})\beta_2] + Var[\phi(\,\cdot\,|_{\text{females}})\beta_2] + Var[\phi(\,\cdot\,|_{\text{females}})\beta_3)] + 2Cov[\phi(\,\cdot\,|_{\text{females}})\beta_2, \phi(\,\cdot\,|_{\text{females}})\beta_3)]}} \sim t_k \quad (1)$$

Where k are the degrees of freedom of a t-distribution using the Satterthwaite approximation.

3. Nonlinear in Q. The models in Equation (2) and (3) of the main article assume a linear relationship between the answer to a question and the position of the question in the test. This is a rather strong assumption, as estimating $y_{hij} = \beta_0 + \beta_1 Q_{ij} + \beta_2 Q_{ij}^2 + \epsilon_{hij}$ does show the presence of nonlinear effects.[10]

Despite the deviations from linear appear to be small and not homogeneous across countries, we also tested whether allowing for nonlinear effects has consequences for the gender difference.[11] First, estimating Equation (2) while adding a quadratic term, $y_{hij} = \beta_0 + \beta_1 F_i + \beta_2 Q_{ij} + \beta_3 Q_{ij} F_i + \beta_4 Q_{ij}^2 + \epsilon_{hij}$, gave us identical results to the baseline results in the main text. Second, we estimated Equation (2) while including an interaction between the quadratic term and the female dummy: $y_{ij} = \beta_0 + \beta_1 F_i + \beta_2 Q_{ij} + \beta_3 Q_{ij} F_i + \beta_4 Q_{ij}^2 + \beta_5 Q_{ij}^2 F_i + \epsilon_{hij}$. The marginal effect of Q in this case equals $\beta_2 + \beta_3 F + 2\beta_4 Q + 2\beta_5 QF$. As such, the relevant test for the gender difference in performance during the test becomes $\beta_3 + 2\beta_5 Q \neq 0$. The difference between males and females depends on the position of the question in the test. We tested for the presence of a gender difference at every possible value of Q, which also provided insides in the distribution

[10]The results for the linear coefficient in Q (β_1) showed the estimate is significantly negative for 63 of the 74 countries at the 5% level. In zero cases it was significantly positive. The quadratic estimate in Q (β_2) was significantly negative (positive) for 55 (3) countries at the 5% level. As such, for most countries the decline in performance increased during the test. Note that for all 74 participating countries we found either a significant negative estimate for β_1 or for β_2.

[11]Supplementary Figure 12 shows the fitted values for a linear and quadratic estimate of the decline in performance for the median country in terms of the nonlinear effect size (Italy). The linear line seems to approximate the quadratic line relatively well. Supplementary Figure 13 visualizes that the exact shape of the decline differs per country, by showing the fitted values of the quadratic performance decline for the five countries with the most extreme nonlinear shapes.

of the gender difference throughout the test. Note that for 27 countries the estimate for β_5 was significantly negative at the 5% level, which implies that for some countries the gender difference in sustaining performance decreases as the test goes on.[12]

Supplementary Figure 14 graphs the number of countries for which the gender difference ($\beta_3 + 2\beta_5 Q$) is significantly different from zero at the 5% level at each position of the test. The black bars indicate females were better able to sustain their performance, whereas the grey bars do this for males. Until halfway the test ($Q = 0.5$) there is strong support that females were better able to sustain their performance. Thereafter the gender difference decreases, but up until the end of the test there are more countries for which females were better able to sustain their performance than males (18 versus 8). We found strong evidence for the gender difference, but the size seems to decrease towards the end of the test.

4. A Relative Measure. One might argue that a relative version of the decline in performance is a more comprehensive measure. Imagine a simple version of Equation (1) in the main article represented by $y = \alpha + \beta Q$. Test takers with gender $A \in \{\text{male}, \text{female}\}$ score 1 at the beginning of the test and $\frac{1}{2}$ at the end of the test, where test takers with gender $B \neq A$ score, respectively, $\frac{1}{2}$ and $\frac{1}{4}$ at the beginning and end of the test. The linear equation representing the probability of a correct answer is $y = 1 - \frac{1}{2}Q$ for gender A and $y = \frac{1}{2} - \frac{1}{4}Q$ for gender B, where the decline in performance is $-\frac{1}{2}$ for A and $-\frac{1}{4}$ for B. However, as for both sexes the score at the end of the test is half the score at the beginning of the test, one might prefer a measure that shows a similar decline in performance. In other words, as gender A started off at a higher level compared to gender B, it is also allowed to have a larger absolute deterioration in performance during the test.[13]

Note that such an alternative relative measure does not have qualitative consequences for our results; females' ability to better sustain their performance is unrelated to whether they score better or worse at the beginning of the test. However, this relative measure might capture why the gender difference in sustaining test performance was slightly more prevalent in math and science compared to reading.

A relative measure can be obtained by computing the ratio between the slope and the constant. Note that by implementing this correction, the above example would exhibit the same decline for A and B, that is: $\frac{\beta_A}{\alpha_A} = \frac{\beta_B}{\alpha_B} = -\frac{1}{2}$. The proposed correction for the complete test can be analyzed by the following nonlinear Wald test:

[12]For the other 47 countries we could not reject the null hypothesis of $\beta_5 = 0$ at the 5% level. When we did not reject the null hypothesis of $\beta_3 = 0$ or $\beta_5 = 0$ we did set the estimate equal to zero while calculating the marginal effects.

[13]A usual way of dealing with this type of concern consists of taking the logarithm of the dependent variable and interpreting the coefficients as a rate rather than as a slope (semi-elasticity). This is not possible in our setup given the presence of zeros in the dependent variable.

$$H_0 : \frac{\beta_2}{\beta_0} = \frac{\beta_2+\beta_3}{\beta_0+\beta_1}$$
$$H_1 : \frac{\beta_2}{\beta_0} \neq \frac{\beta_2+\beta_3}{\beta_0+\beta_1}$$

Like in our baseline results, there are only three countries in which the relative decline in performance was smaller for males and in none of these countries the difference is statistically significant.[14] Moreover, the number of countries for which females were significantly better able to sustain their performance notably increases from the 56 found in our baseline results to 64. This relative measure reinforces our baseline results.

When analyzing gender differences in sustaining performance during the test per topic T we implemented the following test:

$$H_0 : \frac{\gamma_2^T}{\gamma_0^T} = \frac{\gamma_2^T+\gamma_3^T}{\gamma_0^T+\gamma_1^T}$$
$$H_1 : \frac{\gamma_2^T}{\gamma_0^T} \neq \frac{\gamma_2^T+\gamma_3^T}{\gamma_0^T+\gamma_1^T}$$

Using this approach, there are 72 out of 74 countries for which females were better able to sustain their performance in reading, where the statistical significance increases from 36 countries in the main text to 54 countries under this alternative specification. This result follows from the fact that in most countries females experienced a higher performance at the start of the test in reading.

In the case of math and science questions males started off from a higher level. Despite this, the results are very similar to the ones obtained in the main text. The number of countries in which females were significantly better able to sustain their performance is 37, compared to 41 in our baseline results. There is one additional country for which the ability to sustain performance during the test favored males, seven in total. In none of these seven countries the difference was statistically significant.

4.1. Details on Non-Linear Wald Test. As we wanted to test whether students with a higher starting performance also have a larger decline in performance, we specified the following nonlinear test (coefficients from Equation (2)): $\frac{\beta_2}{\beta_0} = \frac{\beta_2+\beta_3}{\beta_0+\beta_1}$. The nonlinear Wald test is invariant to algebraically equivalent ways of writing the nonlinear combinations of coefficients [21]. As such, Cameron and Trivedi suggested testing the combination in multiple algebraically equivalent ways. Our results did not change if we tested the following mathematically equivalent combination of coefficients: $\beta_2(\beta_0 + \beta_1) = \beta_0(\beta_2 + \beta_3)$.

Note that the test on the nonlinear combination of coefficients can be interpreted in terms of whether the ratio $\frac{P[y_{Q=1}]}{P[y_{Q=0}]}$ is equal between males and females, where $P[y_{Q=1}]$ and $P[y_{Q=0}]$ denote the probability of having a correct answer on the last and on the first question of the test, respectively. If we follow Equation (2), we see that this ratio for males equals $\frac{\beta_0+\beta_2}{\beta_0}$ and

[14]The precise results can be found in Supplementary Table 12.

for females this ratio is $\frac{\beta_0+\beta_1+\beta_2+\beta_3}{\beta_0+\beta_1}$. If we want to test whether these ratios are equal, we test: $1 + \frac{\beta_2}{\beta_0} = 1 + \frac{\beta_2+\beta_3}{\beta_0+\beta_1}$. This exactly matches the test that we started with above.

5. Unreached Questions. In our main specification unreached questions were coded as missing. Although on average males had slightly more unreached questions than females (respectively 0.763 and 0.755 unreached questions on a test with roughly 60 questions), one might be worried that our baseline results partially pick up that females spent more time on each question trying to provide an accurate answer. In this section, we investigate the robustness of our findings by considering the case in which unreached questions are coded as wrong answers. The most suitable PISA wave to carry out this analysis is the PISA 2015, because it minimizes possible mistakes in the classification of unreached items.[15] Supplementary Figure 15 documents our results are unchanged.

6. Increasing the Number of Questions to Measure Performance at the Start. Using the performance on the first question as a measure for the gender gaps at the beginning of the test might be too restrictive. At the same time, one might think the decline in performance is not severe at the first items of the test. We test for the robustness of our results by increasing the number of questions that are considered to be at the beginning. We re-estimated Equation (2) and (3) while coding the first five questions as the initial ones by setting a value of $Q_{ij} = 0$ for any item j that was ordered in any of the first five positions in the test. By doing so the results were virtually identical to our baseline results, see Supplementary Figure 16 and 17.

7. Short Break after one Hour. The PISA test takers had a short break of typically 5 minutes after one hour of test taking. We tested whether this short break affects the gender difference by making use of the halfway dummy H and re-estimating Equation (2) as follows: $y_{hij} = \beta_0 + \beta_1 F_i + \beta_2 Q_{ij} + \beta_3 Q_{ij} F_i + \beta_4 H_{ij} + \beta_5 Q_{ij} H_{ij} + \epsilon_{hij}$. We do not know at which item the student exactly was when they were allowed to have the short break, therefore we simply conceptualized H to be equal to 1 if the student was halfway during the test ($Q \geq 0.5$) and 0 otherwise. Supplementary Figure 18 documents the inclusion of this break does not affect the gender difference, by showing that the results are identical to those reported in the main text.

8. Question Type. The PISA uses several types of questions in its assessment. In the PISA 2009 the questions are classified into the following categories: multiple-choice, complex multiple-choice, open constructed response, closed constructed response, short response, and open response. To test whether the question type may have an effect on our results, we separated test items into two groups: multiple choice questions and questions that involve some degree of

[15] Unreached items are defined as all the successive unanswered questions clustered at the end of a test, except for the first missing answer [3]. The possibility of going back and forth across test items makes the pen-and-paper based PISA waves prone to an incorrect categorization of unreached items.

openness in the provided answer (open constructed response, closed constructed response, short response, and open response). In Supplementary Figure 19 and 20 we observe that the gender difference is present and very similar across both types of questions.

Supplementary Note IV: Robustness of Study 2

In this section we investigate the robustness of Study 2. Column (1) of Supplementary Table 9 displays the original estimates of Table 1 in the main text. The constant reveals that on very short tests, males perform 0.2 standard deviations better than females, but females fully close this gap on a test with 125 questions. Table 1 in the main text has already shown three checks to confirm the robustness of this pattern. First, we collected information on the gender gap of the tests, where the results did not change if we consider the gender gap we calculated. Moreover, we excluded one extreme test with 240 questions[16] and gave a weight of one-half to studies that we coded differently than the Lindberg dataset [22]. This did not change our results.

The statistically significant negative association notwithstanding, there does appear to be a lot of noise in the math gender gap as displayed by the low adjusted R^2. A substantial part of the tests in our sample contained few questions; they were the ones that also introduced a large part of the unexplained variance. In columns (2) and (3) of Supplementary Table 9 we trimmed our sample and excluded the exams with fewer than 10 and 40 questions, respectively. The fit increased while excluding shorter tests. Possible explanations for this are that short tests are subject to more noise (when measuring ability) or that the number of questions is a worse proxy for test length when there are fewer questions.

An alternative measure for the length of the test is the maximum time allowed to complete the test. We preferred to use the number of questions to measure test length, because with this measure the results of Study 2 are not necessarily linked to gender differences in performance under time pressure. Indeed, in the main text we used the relationship between the maximum time allowed to complete the test (i.e., the alternative measure) and the number of questions to argue that time pressure is unlikely to explain the findings in Study 2.

Nevertheless, we redid the basic analysis while regressing the math gender gap on a constant and the maximum time allowed to complete the test as a measure of test length. Column (4) of Supplementary Table 9 shows that the maximum time was also negatively associated with the gender gap, but insignificantly so (p-value=0.30, two-sided t-test). We redid the analysis in Table 1 and used our own recalculated gender gap and gave a weight of one-half to tests that we coded differently (columns (5) and (6)). This did not change our results. Next, we trimmed

[16]The second longest test contained 135 questions.

our sample for the same reasons as with the number of questions: to exclude extremely long tests and to remove short tests that might include more noise concerning the measured gender gap. Column (7) removes five extreme tests that took longer than 170 minutes and reports a significant negative estimate for the coefficient of maximum time allowed to complete the test at the 1% level.[17] Columns (8) and (9) also exclude tests with a time limit shorter than 5 and 20 minutes, respectively, and also find significant negative effects.

Next, we investigated whether the relationship between the math gender gap and the length of the test in Study 2 was related to the gender difference in sustaining performance during the test that were found in Study 1. One competing explanation might be that long and short tests in Study 2 simply differ in other characteristics that correlate with the gender gap as well. Columns (2) and (3) of Supplementary Table 10 provide evidence against this competing explanation. By using information on the world region in which the test was given, the columns split the sample into world regions for which the relationship between the math gender gap and number of questions is strongly present (Europe, Australia, and the Middle East) and for which it is not present at all (Asia). Column (1) shows the estimate for the whole sample as a comparison. If gender differences in sustaining performance during the test that were found in Study 1 drive this relationship, we would expect those to be larger in Europe, Australia, and the Middle East than in Asia. Using the baseline results of Study 1 documented in the main text, we observe that the gender difference in decline is indeed two times as small in Asian countries. A regression of the size of the gender difference (β_3) on a dummy that equals 0 if the country is Asian and 1 if its European, Australia, or in the Middle East reveals a significant positive estimate with a t-statistic of 4.00 (robust standard errors).

Supplementary Note V: Low Stakes versus High Stakes

In this section, we elaborate upon the analysis that was briefly presented in the Discussion of the main text to investigate whether the results of Study 1 might also be valid in tests with higher stakes.

Many studies have found the existence of gender differences in performance when under pressure and in competitive environments [23, 24, 25]. Two recent studies showed that females perform relatively worse as the stakes on a test increase [26, 27]. Moreover, males get better test scores when they are competing for college seats than what would be predicted by their previous grades, while the opposite is true for females [28]. In contrast to this latter branch of literature, in our setting the test takers did not face competition or pressure. In fact, final

[17] The sixth longest test had a maximum time of 135 minutes.

scores of the PISA test are not communicated to the test takers.

Is the low stakes nature of the PISA test responsible for the observed gender difference in ability to sustain performance? Given the discussion on the possible determinants in the Results section of the main text, one might expect it to be smaller (or even absent) in a high-stakes context. If this were true, our results might provide an additional explanation to the observation of females performing relatively better on low-stakes tests than on high-stakes ones [26, 27]. In this section we investigate the possible influence of stakes in our results.

First, we tested whether the relationship between the math gender gap and the length of the test in Study 2 was also present on tests with stakes. To do so, we coded whether tests included in the dataset of Study 2 had any stakes. While this information was unavailable for 90 studies, column (4) of Supplementary Table 10 shows that the same negative relationship is found when restricting the regression to tests with stakes.

Secondly, we took advantage of country differences in testing culture. Students in Shanghai have higher test motivation than U.S. students, as in response to financial incentives, performance among Shanghai students did not change, while the test scores of U.S. students increased substantially [29]. Sjøberg argues that institutional promotion and motivational messages regarding international standardized tests are more prevalent in Asian countries and discusses the specific case of Singapore [30]. If the stronger test taking culture found in these previous articles are relevant to the Asian countries participating in the PISA, it could explain the smaller gender difference that we found in Asian countries. Then, higher stakes may reduce the gender differences in ability to sustain performance throughout the test. Note, however, that for 60 percent of the Asian countries, the gender difference in sustaining performance during the test is present and statistically significant. Relating our results to the ones by Sjøberg [30], in both PISA waves in which Singapore participated (2009 and 2012) we found a significantly less steep decline for females (at the 1% level in 2012 and at the 10% level for 2009). With respect to Gneezy and co-authors [29], the PISA 2009 only sampled Chinese test takers from Shanghai. Supplementary Table 5 shows that in Shanghai, males significantly outperform females at the beginning of the test in math and science by more than 3 percentage points, but females significantly reduce the gender gap as the test goes on. The gap is exactly offset at the end of the test. To sum up, by considering cross country differences in testing cultures, we find evidence to suggest that the gender difference in ability to sustain performance is smaller but not absent in the presence of stakes.

Ultimately, we would like to have a measure of motivation for the PISA test per country and study its association with the size of the gender difference in ability to sustain performance. To construct such a measure, we used the average number of unanswered test questions per

student as a measure of test motivation. The idea being that, as the PISA test has no penalty for incorrect answers, not giving an answer to a question is a strictly dominated strategy. We expect that this type of careless testing behavior would occur less often if the perceived stakes were high. We regressed the size of the gender difference in ability to sustain performance on our measure for the stakes of the PISA test. By doing so, we did not find that the gender difference is larger in countries where the incidence of non-response is higher. To the contrary, we found that countries with a low non-response rate (i.e., high subjective stakes) had a somewhat larger gender difference in their ability to sustain performance. Similar to before, this result suggests that the gender difference in sustaining performance throughout the test is not necessarily absent in a high-stakes context.

Supplementary Figures and Tables

Supplementary Figure 1

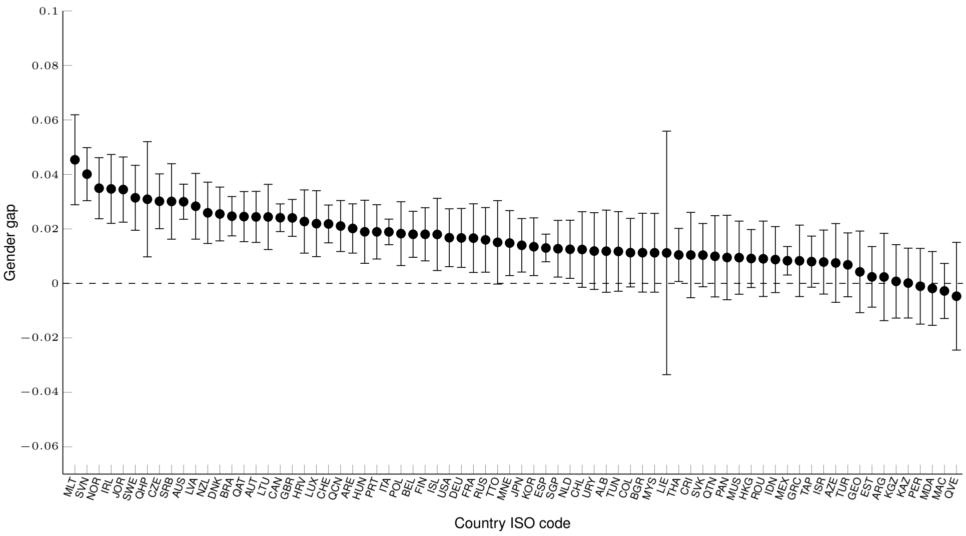

Gender differences in sustaining performance on the cluster level. The figure plots the estimate of the gender difference in sustaining performance during the test for each country participating in the PISA 2009. Positive values indicate countries in which females are better able to sustain their performance during the test than males. Error bars represent the 95 percent confidence intervals. Source data are provided as a Source Data file (Study 1).

Supplementary Figure 2

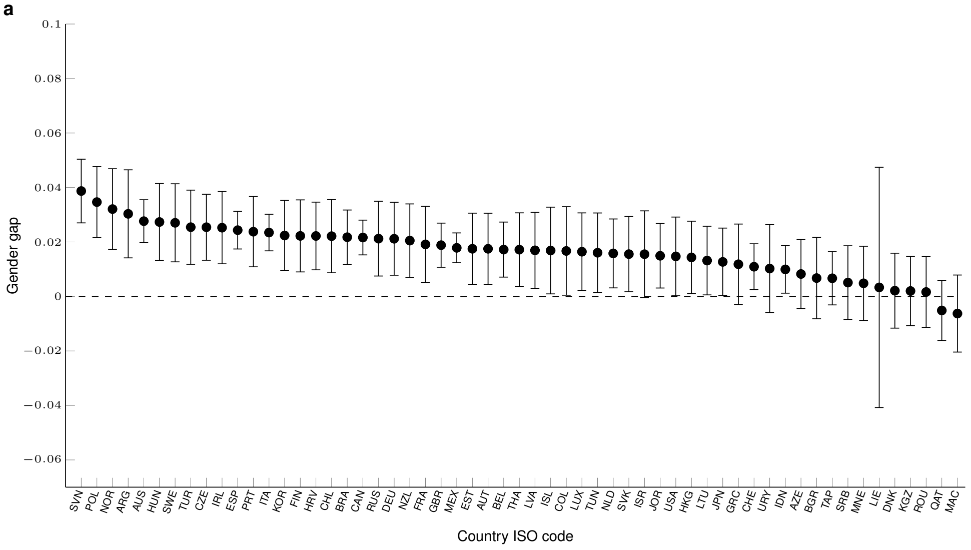

All questions (PISA 2006)

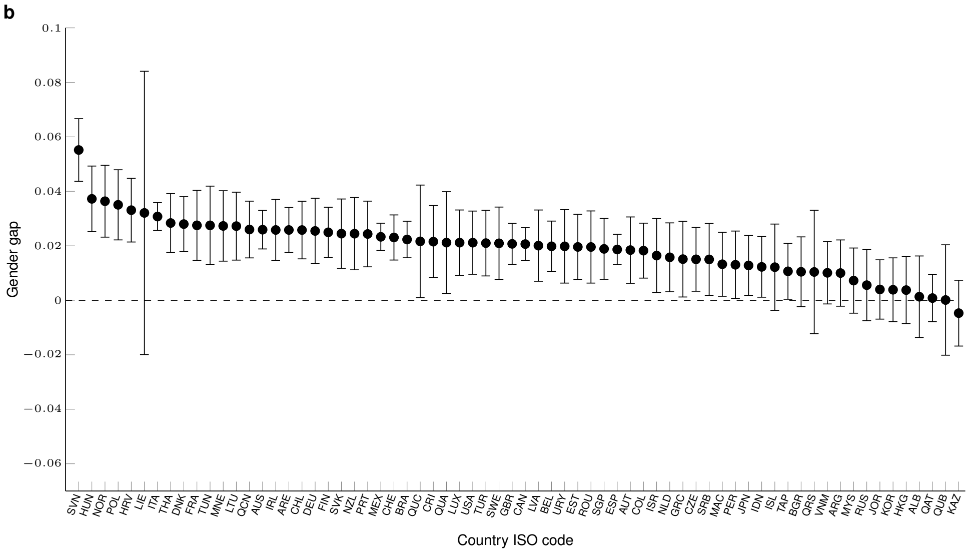

All questions (PISA 2012)

Gender differences in sustaining performance. The figures plot the estimates of the gender difference in sustaining performance during the test for each country participating in (a) the PISA 2006 and (b) the PISA 2012. Positive values indicate countries in which females are better able to sustain their performance during the test than males. Error bars represent the 95 percent confidence intervals. Source data are provided as a Source Data file (Study 1).

Supplementary Figure 3

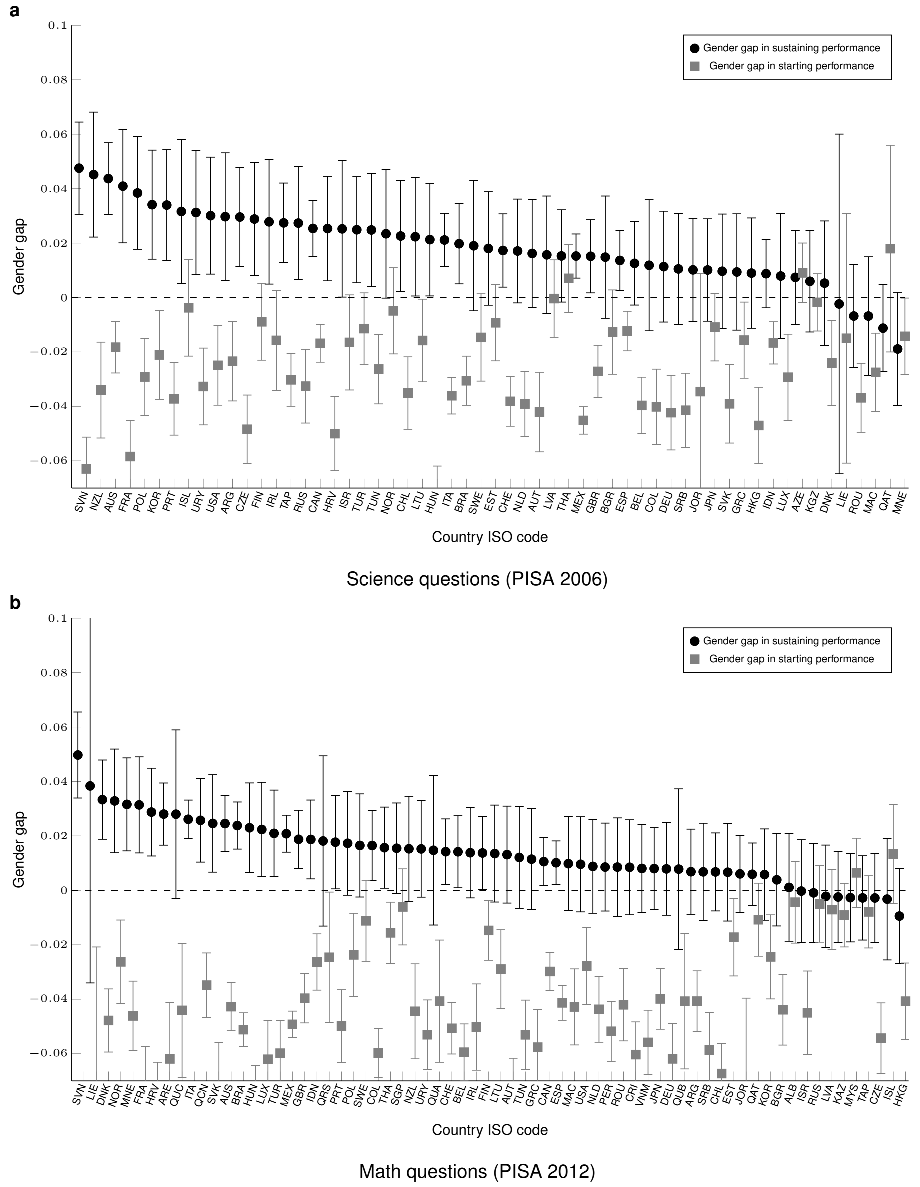

Gender differences in starting performance and in sustaining performance by topic. The figure plots the estimate of the gender gap in starting performance and in sustaining performance during the test for each country participating in (a) the PISA 2006 (science) and (b) the PISA 2012 (math). Positive values indicate the gender gap favors females. Error bars represent the 95 percent confidence intervals. Source data are provided as a Source Data file (Study 1).

Supplementary Figure 4

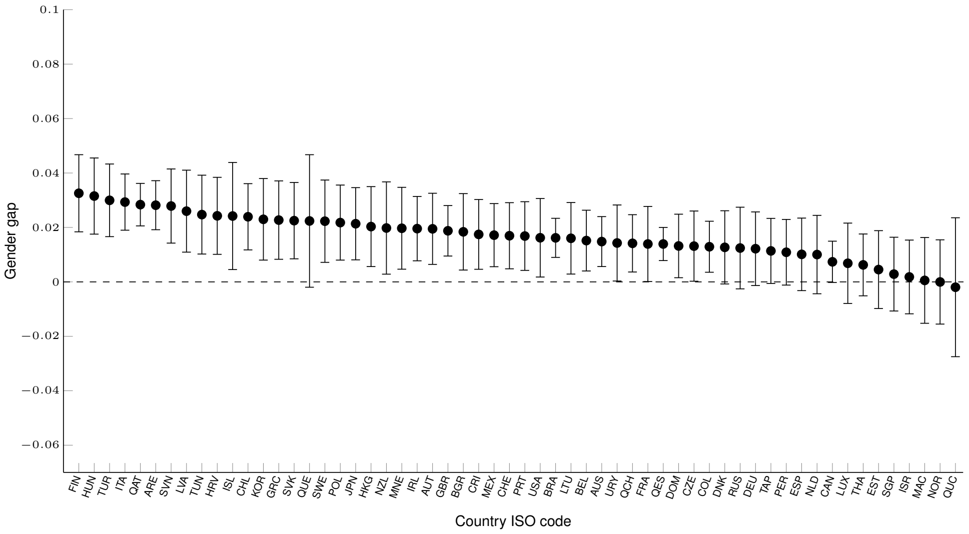

Gender differences in sustaining performance. The figure plots the estimate of the gender difference in sustaining performance during the test for each country participating in the PISA 2015. Positive values indicate countries in which females are better able to sustain their performance during the test than males. Error bars represent the 95 percent confidence intervals. Source data are provided as a Source Data file (Study 1).

Supplementary Figure 5

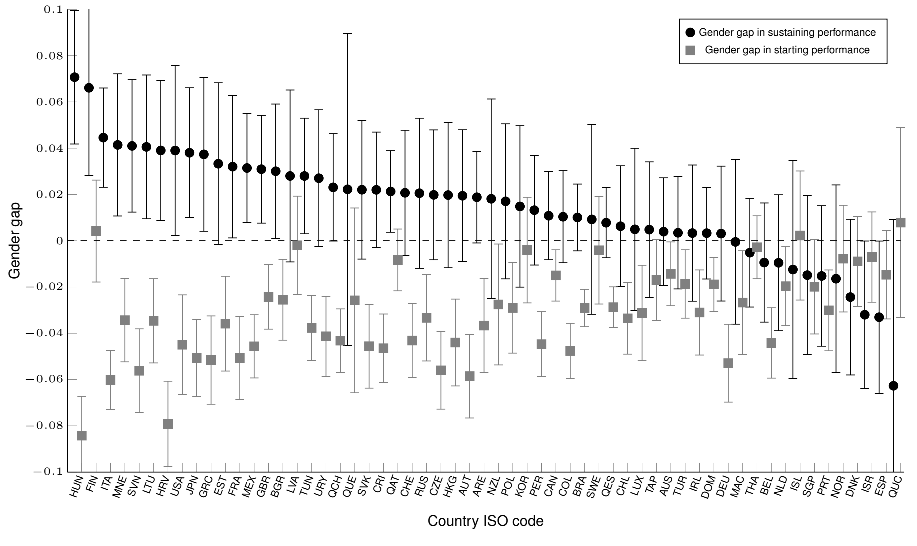

Science questions (PISA 2015)

Gender differences in starting performance and in sustaining performance by topic. The figure plots the estimate of the gender gap in starting performance in science and in sustaining performance during the test in science for each country participating in the PISA 2015. Positive values indicate the gender gap favors females. Error bars represent the 95 percent confidence intervals. Source data are provided as a Source Data file (Study 1).

Supplementary Figure 6

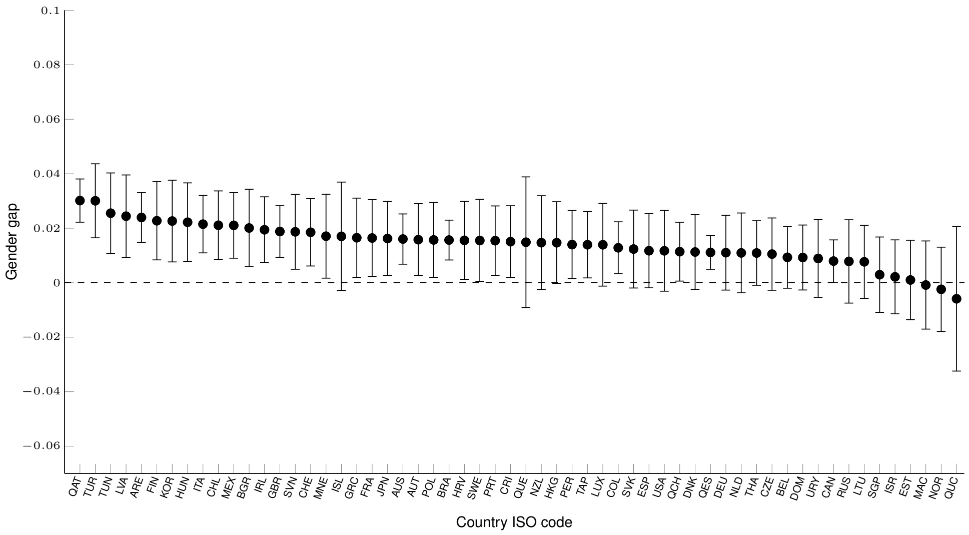

Gender differences in sustaining performance on the unit level. The figure plots the estimate of the gender difference in sustaining performance during the test for each country participating in the PISA 2015. Positive values indicate countries in which females are better able to sustain their performance during the test than males. Error bars represent the 95 percent confidence intervals. Source data are provided as a Source Data file (Study 1).

Supplementary Figure 7

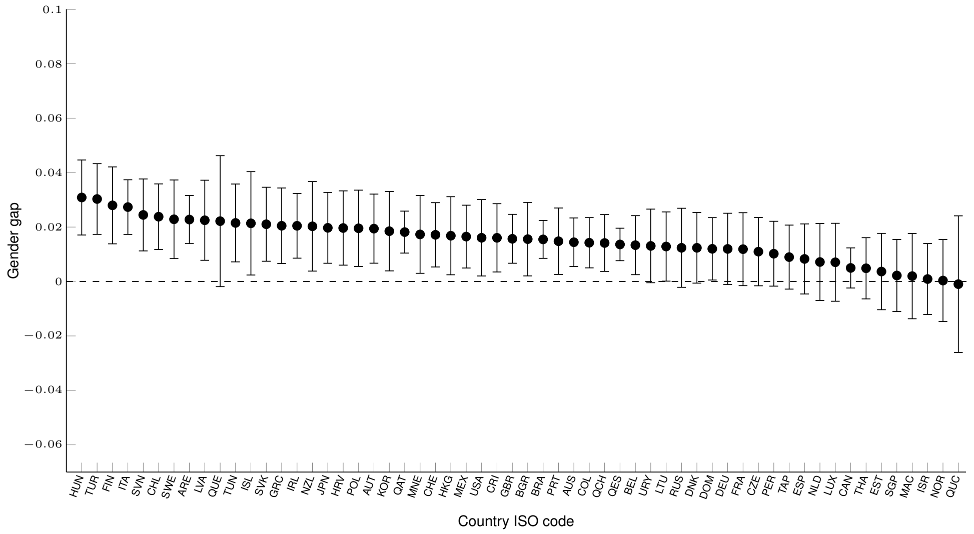

Gender differences in sustaining performance controlling for number of actions and time spent per question. The figures plot the estimates of the gender difference in sustaining performance during the test for each country participating in the PISA 2015. Positive values indicate countries in which females are better able to sustain their performance during the test than males. Error bars represent the 95 percent confidence intervals. Source data are provided as a Source Data file (Study 1).

Supplementary Figure 8

a

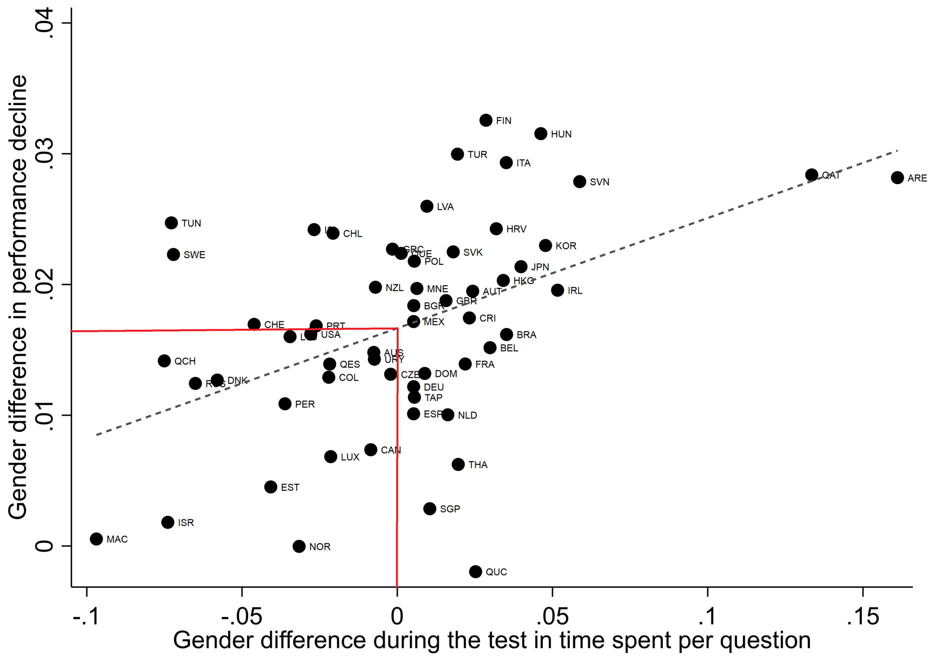

b

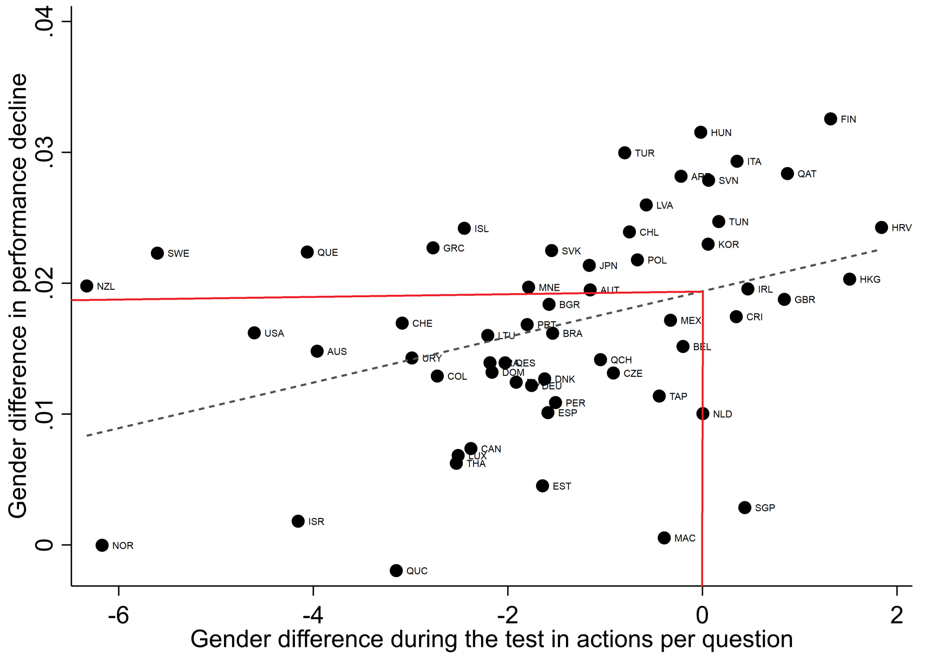

Positive intercept and slope when regressing the gender difference in sustaining performance upon the gender difference in sustaining time spent and number of actions per question during the test. See Supplementary Table 8 for the regression results. The figures are based upon the PISA 2015 and display a scatter plot and a linear OLS regression line between the gender difference in sustaining performance and the gender difference during the test in (a) time spent per question and (b) number of actions per question. Source data are provided as a Source Data file (Study 1).

Supplementary Figure 9

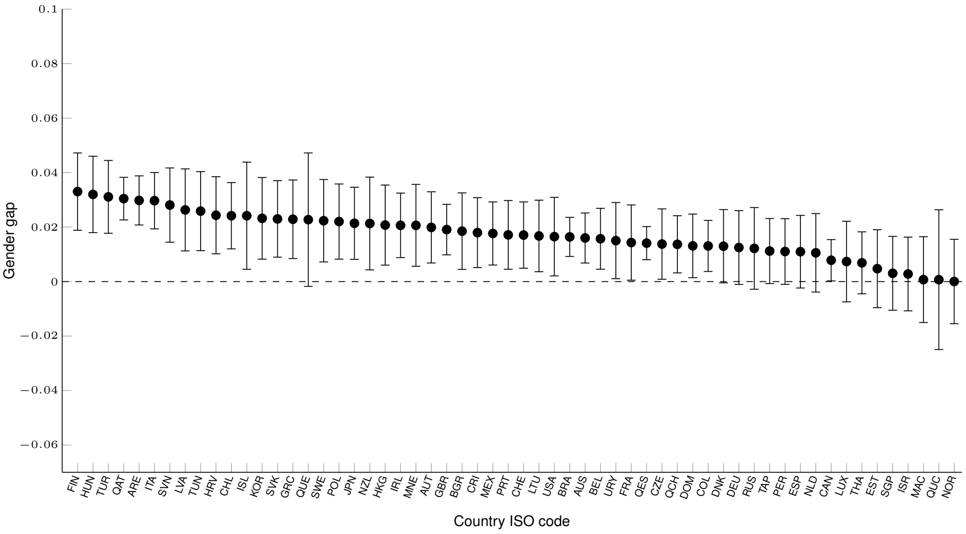

Gender differences in sustaining performance controlling for stumping. The figure plots the estimate of the gender difference in sustaining performance during the test for each country participating in the PISA 2015. Positive values indicate countries in which females are better able to sustain their performance during the test than males. Error bars represent the 95 percent confidence intervals. Source data are provided as a Source Data file (Study 1).

Supplementary Figure 10

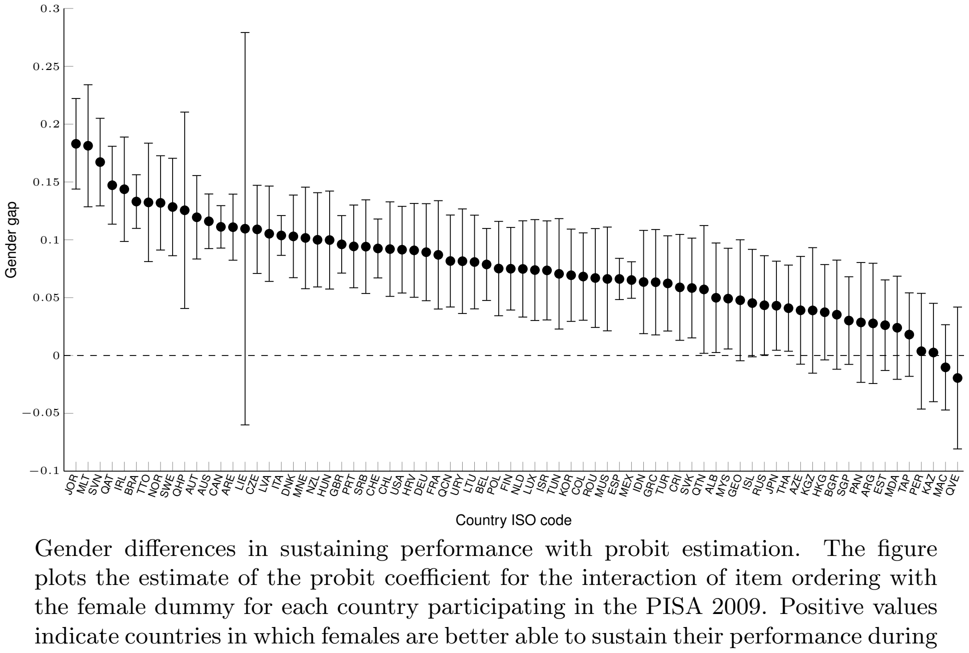

Gender differences in sustaining performance with probit estimation. The figure plots the estimate of the probit coefficient for the interaction of item ordering with the female dummy for each country participating in the PISA 2009. Positive values indicate countries in which females are better able to sustain their performance during the test than males. Error bars represent the 95 percent confidence intervals. Source data are provided as a Source Data file (Study 1).

Supplementary Figure 11

a

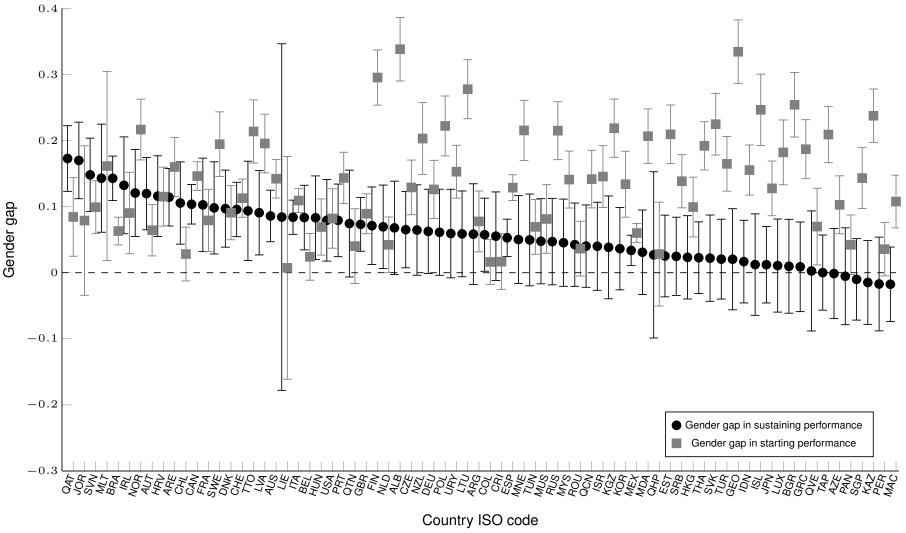

Reading questions

b

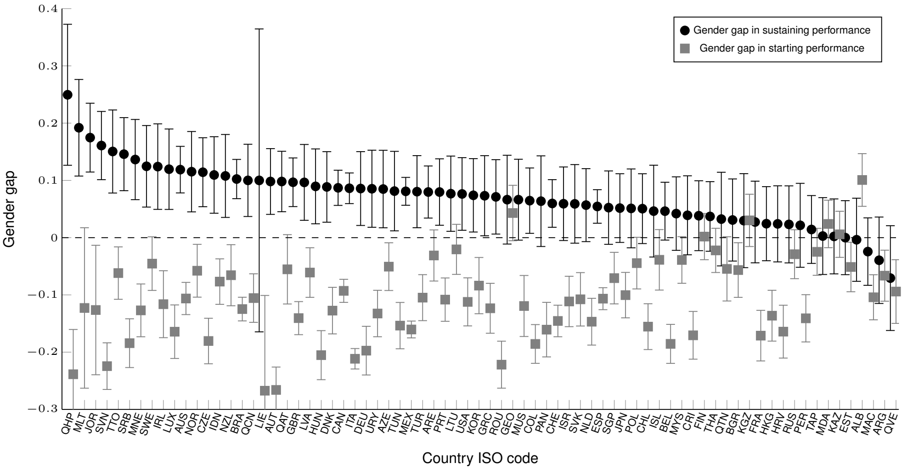

Math-science questions

Gender differences in starting performance and in sustaining performance by topic with probit estimation. The figures plot the estimates of the probit coefficients for the female dummy and the interaction of item ordering with the female dummy for each country participating in the PISA 2009 for (a) reading and (b) math-and-science. Positive values indicate the gender gap favors females. Error bars represent the 95 percent confidence intervals. Source data are provided as a Source Data file (Study 1).

Supplementary Figure 12

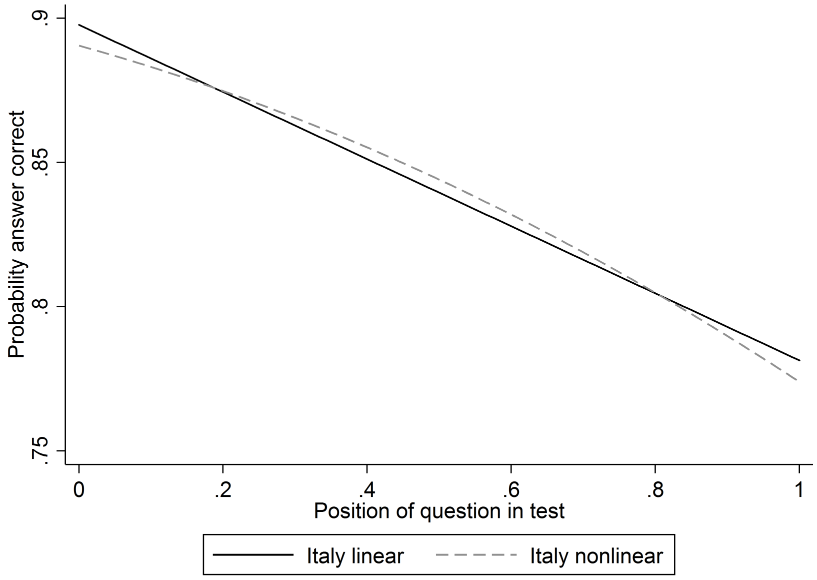

Linear and nonlinear estimate of the decline in performance for Italy. The figure is based upon the PISA 2009 and displays fitted values for a linear (solid line) and quadratic (dashed line) estimate of the performance decline. Source data are provided as a Source Data file (Study 1).

Supplementary Figure 13

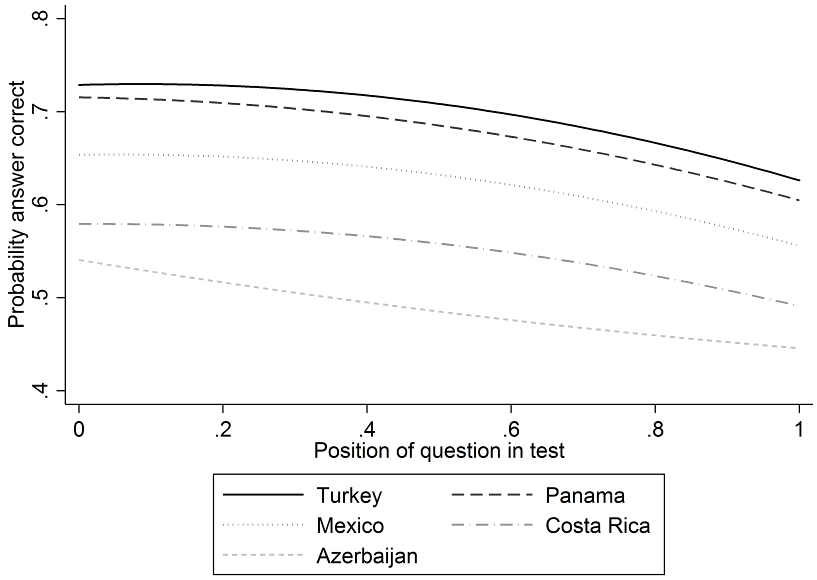

Nonlinear estimates of the decline in performance for the five countries with the most extreme nonlinear shape. The figure is based upon the PISA 2009 and displays fitted values for the quadratic estimate of the performance decline. For Turkey, Mexico, Panama, and Costa Rica the decline increases as the test continues, where the opposite is true for Azerbaijan. Source data are provided as a Source Data file (Study 1).

Supplementary Figure 14

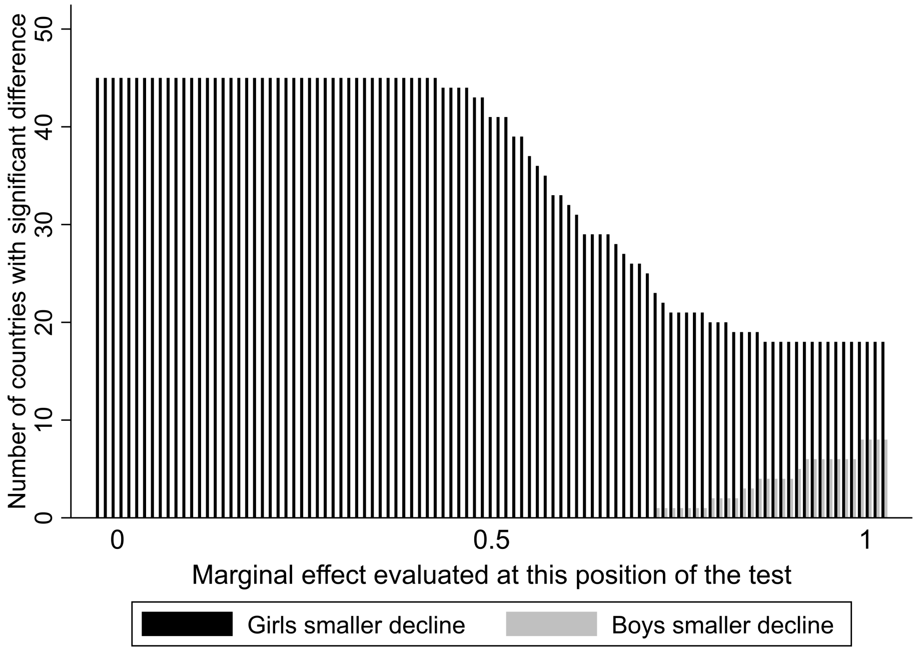

Gender differences in sustaining performance at different positions of the test. The figure displays the number of countries participating in the PISA 2009 for which the gender difference in sustaining performance during the test is significantly different at each position of the test (at the 5% level). Source data are provided as a Source Data file (Study 1).

Supplementary Figure 15

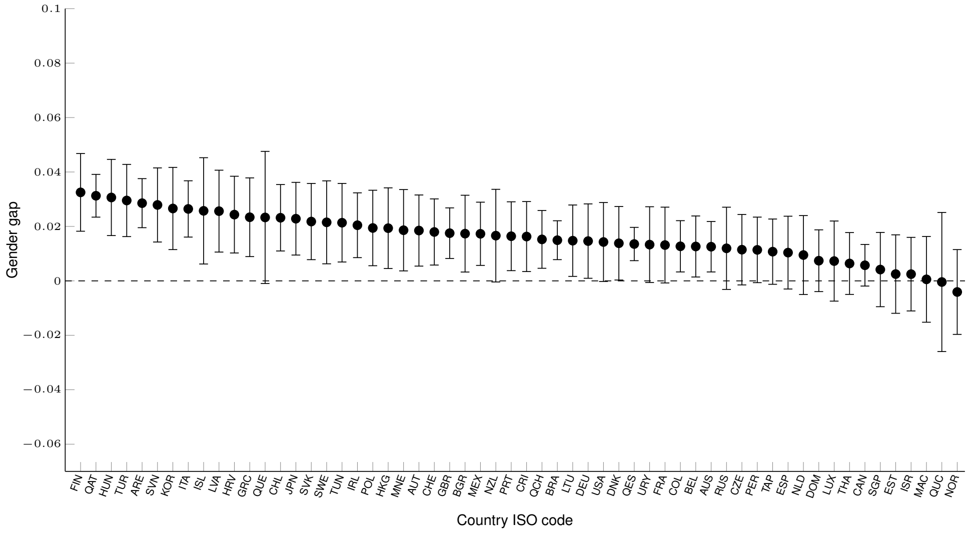

Gender differences in sustaining performance coding unreached questions as wrong. The figure plots the estimate of the gender difference in sustaining performance during the test for each country participating in the PISA 2015. Positive values indicate countries in which females are better able to sustain their performance during the test than males. Error bars represent the 95 percent confidence intervals. Source data are provided as a Source Data file (Study 1).

Supplementary Figure 16

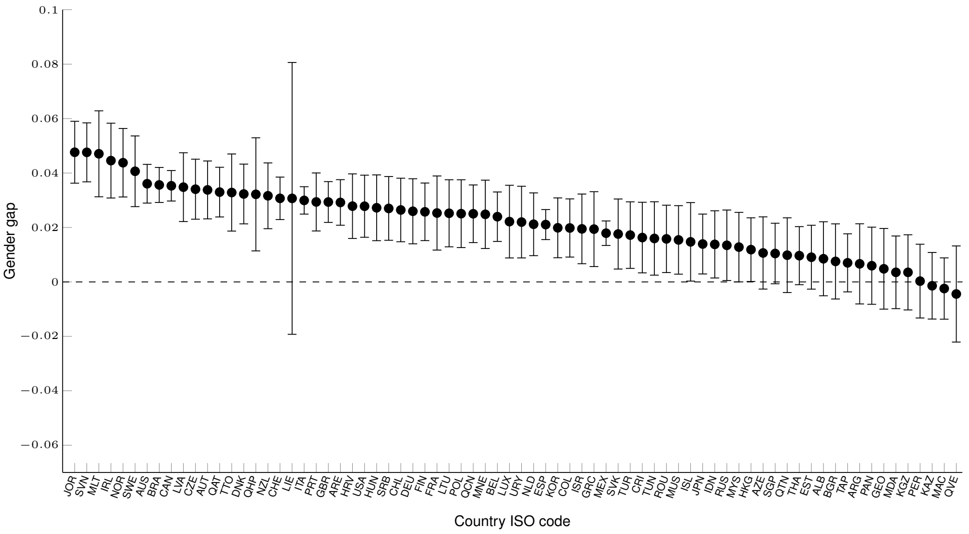

Gender differences in sustaining performance with the first five questions coded as $Q = 0$. The figure plots the estimate of the gender difference in sustaining performance during the test for each country participating in the PISA 2009. Positive values indicate countries in which females are better able to sustain their performance during the test than males. Error bars represent the 95 percent confidence intervals. Source data are provided as a Source Data file (Study 1).

Supplementary Figure 17

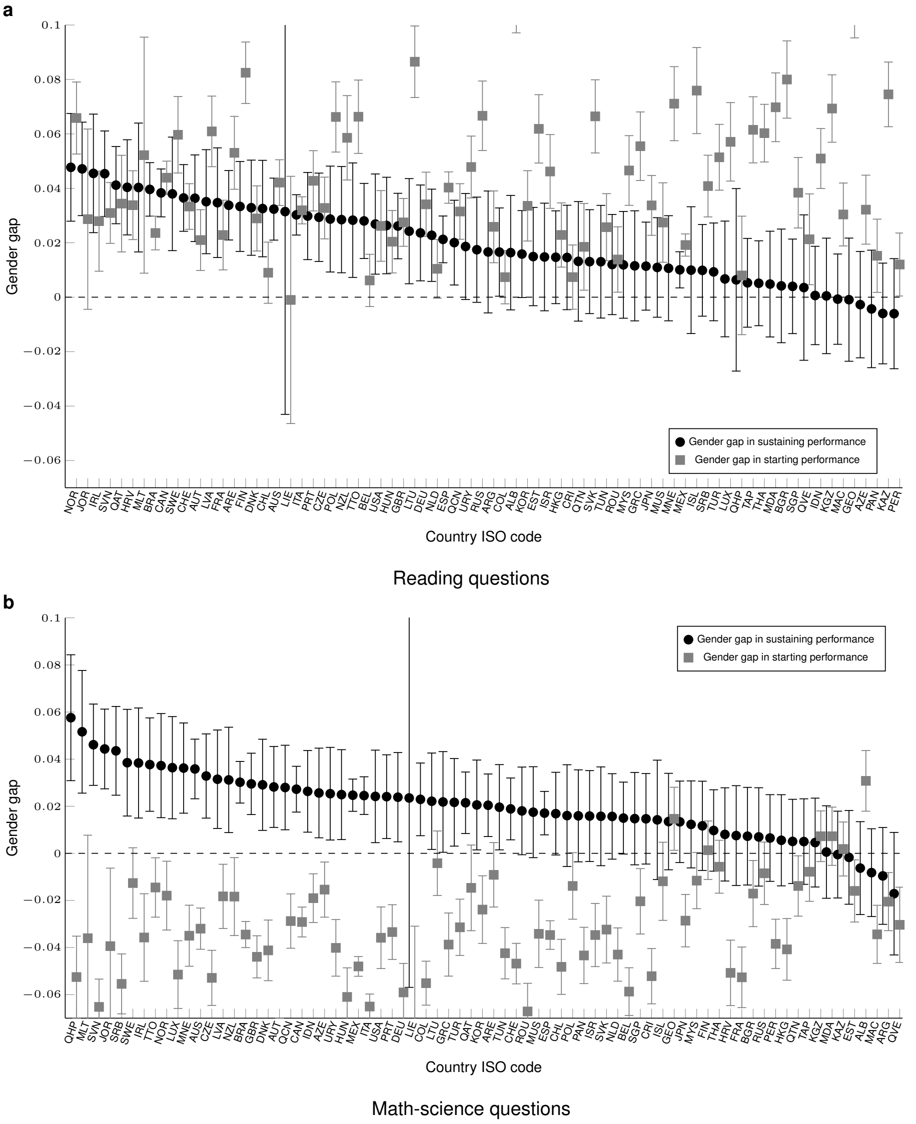

Gender differences in starting performance and in sustaining performance by topic with the first five questions coded as $Q = 0$. The figures plot the estimates of the gender gap in starting performance and in sustaining performance during the test for each country participating in the PISA 2009 for (a) reading and (b) math-and-science. Positive values indicate the gender gap favors females. Error bars represent the 95 percent confidence intervals. Source data are provided as a Source Data file (Study 1).

Supplementary Figure 18

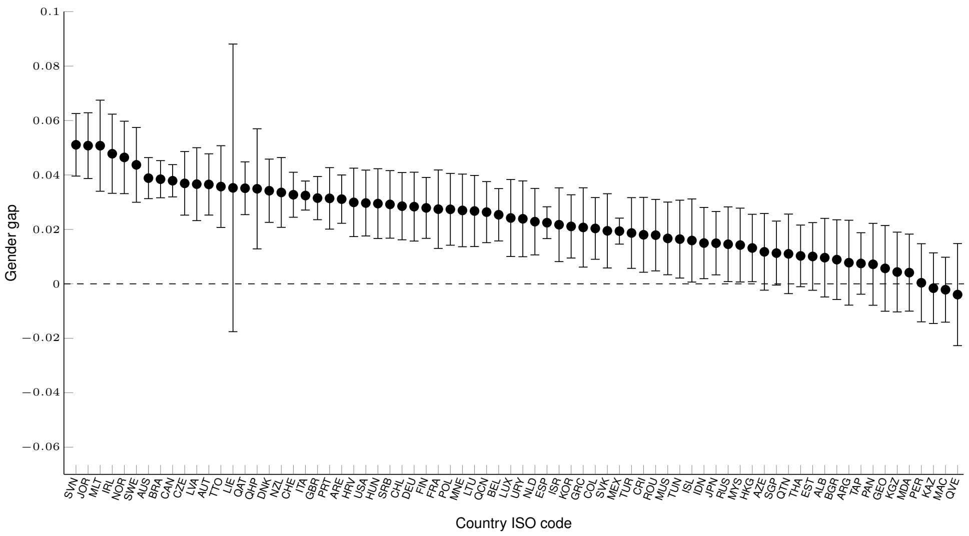

Gender differences in sustaining performance controlling for the short break after one hour. The figure plots the estimate of the gender difference in sustaining performance during the test for each country participating in the PISA 2009. Positive values indicate countries in which females are better able to sustain their performance during the test than males. Error bars represent the 95 percent confidence intervals. Source data are provided as a Source Data file (Study 1).

Supplementary Figure 19

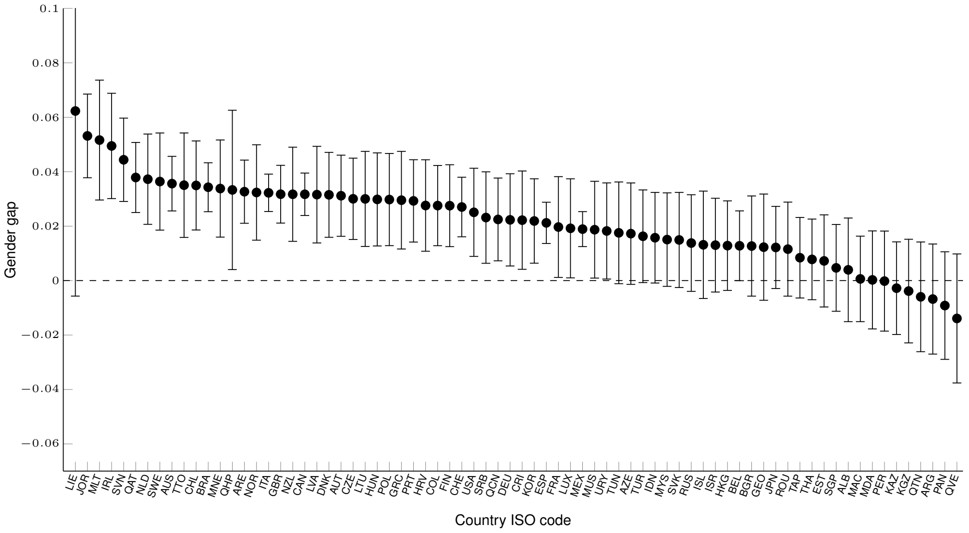

Gender differences in sustaining performance for multiple-choice questions. The figure plots the estimate of the gender difference in sustaining performance during the test for each country participating in the PISA 2009. Positive values indicate countries in which females are better able to sustain their performance during the test than males. Error bars represent the 95 percent confidence intervals. Source data are provided as a Source Data file (Study 1).

Supplementary Figure 20

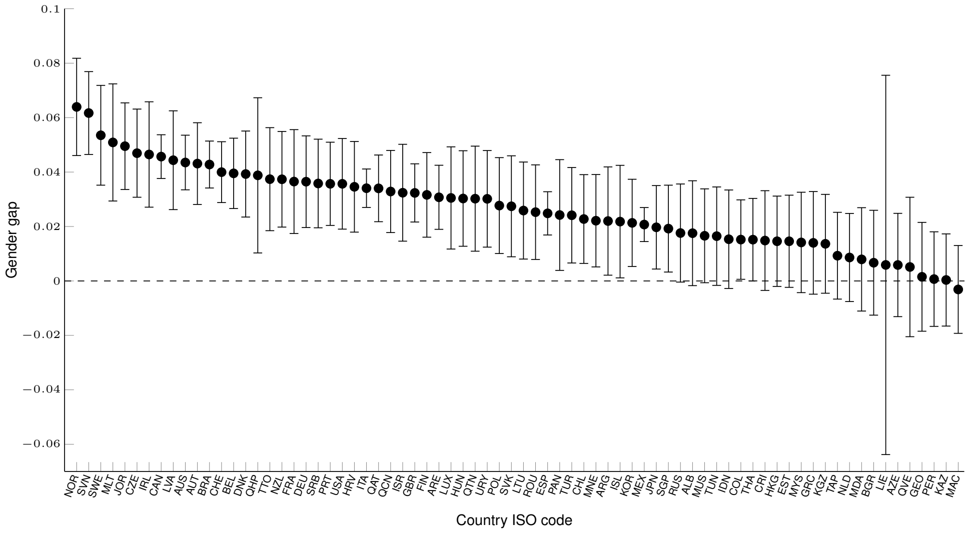

Gender differences in sustaining performance for open-ended questions. The figure plots the estimate of the gender difference in sustaining performance during the test for each country participating in the PISA 2009. Positive values indicate countries in which females are better able to sustain their performance during the test than males. Error bars represent the 95 percent confidence intervals. Source data are provided as a Source Data file (Study 1).

Supplementary Table 1: Gender differences in sustaining performance during the test. The PISA 2009.

Country	Gender diff. during the test	p-value	Country	Gender diff. during the test	p-value	Country	Gender diff. during the test	p-value
ALB	0.0097 (0.0074)	0.1887	HRV	0.0299 (0.0064)	3.06e-06	NZL	0.0335 (0.0065)	2.96e-07
ARE	0.0311 (0.0045)	6.60e-12	HUN	0.0295 (0.0065)	6.50e-06	PAN	0.0070 (0.0077)	0.3622
ARG	0.0076 (0.0080)	0.3384	IDN	0.0150 (0.0067)	0.0250	PER	0.0005 (0.0073)	0.9437
AUS	0.0388 (0.0038)	0.0000	IRL	0.0476 (0.0074)	1.43e-10	POL	0.0275 (0.0067)	0.0000
AUT	0.0365 (0.0057)	2.09e-10	ISL	0.0160 (0.0078)	0.0398	PRT	0.0315 (0.0058)	4.62e-08
AZE	0.0117 (0.0072)	0.1026	ISR	0.0218 (0.0069)	0.0016	QAT	0.0352 (0.0049)	1.09e-12
BEL	0.0254 (0.0049)	2.30e-07	ITA	0.0325 (0.0027)	0.0000	QCN	0.0263 (0.0057)	4.17e-06
BGR	0.0089 (0.0075)	0.2335	JOR	0.0506 (0.0062)	2.22e-16	QHP	0.0349 (0.0113)	0.0019
BRA	0.0385 (0.0035)	0.0000	JPN	0.0150 (0.0059)	0.0118	QTN	0.0110 (0.0075)	0.1396
CAN	0.0379 (0.0030)	0.0000	KAZ	-0.0016(0.0066)	0.8148	QVE	-0.0039(0.0096)	0.6798
CHE	0.0328 (0.0042)	7.99e-15	KGZ	0.0043 (0.0075)	0.5639	ROU	0.0179 (0.0067)	0.0075
CHL	0.0285 (0.0063)	5.97e-06	KOR	0.0211 (0.0059)	0.0004	RUS	0.0147 (0.0070)	0.0358
COL	0.0212 (0.0058)	0.0002	LIE	0.0354 (0.0270)	0.1895	SGP	0.0113 (0.0060)	0.0599
CRI	0.0182 (0.0070)	0.0095	LTU	0.0267 (0.0067)	0.0001	SRB	0.0292 (0.0063)	4.00e-06
CZE	0.0368 (0.0060)	6.17e-10	LUX	0.0242 (0.0072)	0.0008	SVK	0.0195 (0.0070)	0.0050
DEU	0.0284 (0.0065)	0.0000	LVA	0.0366 (0.0068)	8.33e-08	SVN	0.0510 (0.0059)	0.0000
DNK	0.0342 (0.0059)	7.85e-09	MAC	-0.0020(0.0061)	0.7423	SWE	0.0437 (0.0070)	4.63e-10
ESP	0.0226 (0.0030)	3.77e-14	MDA	0.0041 (0.0072)	0.5685	TAP	0.0075 (0.0058)	0.1944
EST	0.0101 (0.0063)	0.1110	MEX	0.0196 (0.0024)	8.88e-16	THA	0.0103 (0.0058)	0.0738
FIN	0.0279 (0.0057)	1.01e-06	MLT	0.0507 (0.0085)	2.94e-09	TTO	0.0356 (0.0077)	3.28e-06
FRA	0.0274 (0.0074)	0.0002	MNE	0.0270 (0.0068)	0.0001	TUN	0.0170 (0.0073)	0.0200
GBR	0.0315 (0.0041)	8.22e-15	MUS	0.0167 (0.0068)	0.0143	TUR	0.0188 (0.0066)	0.0044
GEO	0.0058 (0.0080)	0.4721	MYS	0.0142 (0.0069)	0.0406	URY	0.0241 (0.0071)	0.0007
GRC	0.0208 (0.0074)	0.0050	NLD	0.0229 (0.0062)	0.0002	USA	0.0297 (0.0062)	1.53e-06
HKG	0.0132 (0.0063)	0.0370	NOR	0.0465 (0.0068)	7.79e-12			

Notes: Obtained by OLS estimations of Equation (2). Standard errors (in parentheses) clustered at the student level. The p-values are obtained from a two-sided t-test. Source data are provided as a Source Data file (Study 1).

Supplementary Table 2: Gender differences in starting performance and in sustaining performance during the test by topic. The PISA 2009.

Country	Diff. in reading starting level	p-value	Diff. in reading during the test	p-value	Diff. in science and math starting level	p-value	Diff. in science and math during the test	p-value
ALB	0.1104 (0.0078)	0.0000	0.0176 (0.0114)	0.1213	0.0308 (0.0071)	0.0000	-0.0059 (0.0107)	0.5840
ARE	0.0507 (0.0070)	4.28e-13	0.0359 (0.0069)	1.81e-07	-0.0104 (0.0072)	0.1460	0.0215 (0.0072)	0.0028
ARG	0.0244 (0.0072)	0.0007	0.0185 (0.0121)	0.1251	-0.0199 (0.0068)	0.0034	-0.0102 (0.0111)	0.3619
AUS	0.0400 (0.0045)	0.0000	0.0343 (0.0061)	2.42e-08	-0.0348 (0.0047)	1.71e-13	0.0389 (0.0068)	1.29e-08
AUT	0.0185 (0.0060)	0.0021	0.0387 (0.0086)	6.51e-06	-0.0841 (0.0064)	0.0000	0.0307 (0.0093)	0.0009
AZE	0.0321 (0.0070)	3.91e-06	-0.0025(0.0106)	0.8156	-0.0173 (0.0065)	0.0075	0.0277 (0.0103)	0.0074
BEL	0.0044 (0.0052)	0.3951	0.0295 (0.0074)	0.0001	-0.0597 (0.0055)	0.0000	0.0158 (0.0083)	0.0557
BGR	0.0791 (0.0077)	0.0000	0.0055 (0.0113)	0.6229	-0.0177 (0.0076)	0.0205	0.0080 (0.0112)	0.4755
BRA	0.0207 (0.0034)	8.20e-10	0.0425 (0.0053)	1.11e-15	-0.0368 (0.0031)	0.0000	0.0328 (0.0048)	7.59e-12
CAN	0.0413 (0.0033)	0.0000	0.0407 (0.0047)	0.0000	-0.0312 (0.0034)	0.0000	0.0294 (0.0053)	2.29e-08
CHE	0.0310 (0.0045)	7.83e-12	0.0385 (0.0066)	5.82e-09	-0.0482 (0.0047)	0.0000	0.0203 (0.0071)	0.0043
CHL	0.0068 (0.0061)	0.2626	0.0346 (0.0095)	0.0003	-0.0496 (0.0064)	6.88e-15	0.0186 (0.0095)	0.0509
COL	0.0064 (0.0053)	0.2325	0.0174 (0.0087)	0.0468	-0.0568 (0.0051)	0.0000	0.0247 (0.0084)	0.0032
CRI	0.0061 (0.0064)	0.3468	0.0162 (0.0104)	0.1169	-0.0535 (0.0065)	2.22e-16	0.0164 (0.0104)	0.1152
CZE	0.0306 (0.0061)	5.89e-07	0.0317 (0.0087)	0.0003	-0.0554 (0.0064)	0.0000	0.0355 (0.0097)	0.0002
DEU	0.0324 (0.0064)	4.44e-07	0.0254 (0.0095)	0.0075	-0.0612 (0.0067)	0.0000	0.0264 (0.0102)	0.0100
DNK	0.0269 (0.0064)	0.0000	0.0346 (0.0094)	0.0002	-0.0431 (0.0070)	6.71e-10	0.0308 (0.0105)	0.0032
ESP	0.0389 (0.0031)	0.0000	0.0225 (0.0046)	1.24e-06	-0.0360 (0.0033)	0.0000	0.0184 (0.0050)	0.0002
EST	0.0609 (0.0068)	0.0000	0.0157 (0.0098)	0.1074	-0.0163 (0.0072)	0.0234	-0.0010 (0.0108)	0.9268
FIN	0.0803 (0.0061)	0.0000	0.0353 (0.0089)	0.0001	0.0001 (0.0067)	0.9868	0.0133 (0.0102)	0.1932
FRA	0.0204 (0.0070)	0.0034	0.0371 (0.0109)	0.0007	-0.0534 (0.0070)	3.02e-14	0.0085 (0.0115)	0.4561
GBR	0.0260 (0.0047)	3.44e-08	0.0273 (0.0065)	0.0000	-0.0464 (0.0048)	0.0000	0.0323 (0.0070)	4.45e-06
GEO	0.1093 (0.0079)	0.0000	0.0000 (0.0122)	0.9974	0.0138 (0.0073)	0.0607	0.0143 (0.0111)	0.1971
GRC	0.0547 (0.0068)	1.33e-15	0.0122 (0.0109)	0.2618	-0.0404 (0.0073)	3.64e-08	0.0235 (0.0116)	0.0421
HKG	0.0218 (0.0064)	0.0006	0.0158 (0.0092)	0.0855	-0.0415 (0.0071)	4.60e-09	0.0067 (0.0105)	0.5231
HRV	0.0311 (0.0068)	4.85e-06	0.0429 (0.0094)	5.59e-06	-0.0515 (0.0075)	8.52e-12	0.0089 (0.0107)	0.4046
HUN	0.0183 (0.0062)	0.0033	0.0286 (0.0096)	0.0028	-0.0628 (0.0068)	0.0000	0.0267 (0.0103)	0.0097
IDN	0.0506 (0.0061)	0.0000	0.0014 (0.0097)	0.8868	-0.0207 (0.0057)	0.0003	0.0277 (0.0094)	0.0033
IRL	0.0251 (0.0097)	0.0100	0.0479 (0.0118)	0.0000	-0.0387 (0.0099)	0.0001	0.0416 (0.0126)	0.0010
ISL	0.0752 (0.0085)	0.0000	0.0105 (0.0126)	0.4021	-0.0131 (0.0090)	0.1443	0.0157 (0.0137)	0.2521
ISR	0.0448 (0.0073)	9.50e-10	0.0167 (0.0107)	0.1176	-0.0362 (0.0073)	6.11e-07	0.0176 (0.0104)	0.0912
ITA	0.0296 (0.0027)	0.0000	0.0328 (0.0040)	2.22e-16	-0.0669 (0.0029)	0.0000	0.0265 (0.0043)	9.37e-10
JOR	0.0256 (0.0170)	0.1336	0.0501 (0.0093)	6.86e-08	-0.0421 (0.0170)	0.0134	0.0468 (0.0092)	3.48e-07
JPN	0.0330 (0.0059)	2.48e-08	0.0121 (0.0087)	0.1656	-0.0296 (0.0060)	8.43e-07	0.0145 (0.0093)	0.1209
KAZ	0.0751 (0.0065)	0.0000	-0.0067(0.0100)	0.5041	0.0018 (0.0063)	0.7692	-0.0005 (0.0100)	0.9617
KGZ	0.0693 (0.0068)	0.0000	0.0005 (0.0115)	0.9685	0.0065 (0.0060)	0.2776	0.0059 (0.0102)	0.5616
KOR	0.0328 (0.0068)	1.63e-06	0.0163 (0.0086)	0.0578	-0.0255 (0.0077)	0.0009	0.0223 (0.0103)	0.0303
LIE	-0.0042(0.0248)	0.8666	0.0357 (0.0401)	0.3732	-0.0833 (0.0271)	0.0021	0.0281 (0.0435)	0.5178
LTU	0.0850 (0.0071)	0.0000	0.0256 (0.0104)	0.0143	-0.0055 (0.0074)	0.4562	0.0233 (0.0111)	0.0354
LUX	0.0563 (0.0078)	4.40e-13	0.0078 (0.0115)	0.4965	-0.0541 (0.0078)	4.14e-12	0.0390 (0.0117)	0.0009
LVA	0.0589 (0.0070)	0.0000	0.0366 (0.0103)	0.0004	-0.0202 (0.0074)	0.0065	0.0331 (0.0113)	0.0034
MAC	0.0302 (0.0062)	1.26e-06	-0.0003(0.0089)	0.9710	-0.0341 (0.0067)	3.45e-07	-0.0082 (0.0101)	0.4171
MDA	0.0691 (0.0069)	0.0000	0.0058 (0.0105)	0.5781	0.0073 (0.0068)	0.2805	0.0003 (0.0107)	0.9757
MEX	0.0183 (0.0022)	2.22e-16	0.0111 (0.0036)	0.0018	-0.0499 (0.0023)	0.0000	0.0267 (0.0037)	6.39e-13
MLT	0.0491 (0.0223)	0.0276	0.0437 (0.0128)	0.0006	-0.0395 (0.0225)	0.0793	0.0550 (0.0141)	0.0001
MNE	0.0703 (0.0074)	0.0000	0.0115 (0.0105)	0.2732	-0.0377 (0.0071)	9.88e-08	0.0392 (0.0104)	0.0002
MUS	0.0265 (0.0078)	0.0007	0.0120 (0.0099)	0.2262	-0.0353 (0.0077)	4.40e-06	0.0186 (0.0105)	0.0755
MYS	0.0457 (0.0070)	5.63e-11	0.0128 (0.0106)	0.2279	-0.0128 (0.0065)	0.0503	0.0139 (0.0100)	0.1667
NLD	0.0088 (0.0059)	0.1372	0.0245 (0.0091)	0.0075	-0.0441 (0.0062)	1.08e-12	0.0169 (0.0098)	0.0847
NOR	0.0625 (0.0071)	0.0000	0.0509 (0.0107)	1.80e-06	-0.0201 (0.0079)	0.0113	0.0389 (0.0120)	0.0012
NZL	0.0570 (0.0082)	3.74e-12	0.0295 (0.0105)	0.0051	-0.0207 (0.0088)	0.0195	0.0336 (0.0121)	0.0055
PAN	0.0152 (0.0074)	0.0396	-0.0038(0.0117)	0.7418	-0.0446 (0.0066)	1.35e-11	0.0174 (0.0106)	0.1009
PER	0.0122 (0.0064)	0.0544	-0.0059(0.0109)	0.5865	-0.0388 (0.0058)	2.02e-11	0.0067 (0.0100)	0.5009
POL	0.0639 (0.0069)	0.0000	0.0313 (0.0105)	0.0028	-0.0152 (0.0077)	0.0475	0.0176 (0.0117)	0.1321
PRT	0.0406 (0.0060)	1.07e-11	0.0320 (0.0087)	0.0002	-0.0350 (0.0063)	2.95e-08	0.0256 (0.0096)	0.0077
QAT	0.0314 (0.0092)	0.0006	0.0442 (0.0077)	8.76e-09	-0.0159 (0.0094)	0.0917	0.0224 (0.0071)	0.0016
QCN	0.0304 (0.0056)	4.55e-08	0.0208 (0.0084)	0.0133	-0.0306 (0.0063)	1.16e-06	0.0296 (0.0097)	0.0022
QHP	0.0070 (0.0120)	0.5623	0.0081 (0.0182)	0.6572	-0.0564 (0.0095)	3.23e-09	0.0614 (0.0145)	0.0000
QTN	0.0174 (0.0087)	0.0442	0.0143 (0.0119)	0.2312	-0.0146 (0.0070)	0.0369	0.0061 (0.0097)	0.5294
QVE	0.0210 (0.0091)	0.0208	0.0039 (0.0144)	0.7854	-0.0297 (0.0088)	0.0007	-0.0170 (0.0141)	0.2271
ROU	0.0125 (0.0066)	0.0574	0.0138 (0.0099)	0.1656	-0.0688 (0.0066)	0.0000	0.0201 (0.0101)	0.0462
RUS	0.0655 (0.0069)	0.0000	0.0184 (0.0104)	0.0777	-0.0092 (0.0072)	0.2022	0.0080 (0.0113)	0.4767
SGP	0.0381 (0.0069)	3.49e-08	0.0043 (0.0094)	0.6442	-0.0215 (0.0075)	0.0040	0.0159 (0.0106)	0.1345
SRB	0.0399 (0.0061)	6.88e-11	0.0110 (0.0091)	0.2242	-0.0586 (0.0069)	0.0000	0.0467 (0.0102)	4.97e-06
SVK	0.0652 (0.0073)	0.0000	0.0146 (0.0103)	0.1562	-0.0337 (0.0077)	0.0000	0.0173 (0.0114)	0.1274
SVN	0.0276 (0.0060)	4.64e-06	0.0488 (0.0085)	9.34e-09	-0.0683 (0.0064)	0.0000	0.0491 (0.0094)	1.51e-07
SWE	0.0570 (0.0076)	4.37e-14	0.0406 (0.0112)	0.0003	-0.0155 (0.0081)	0.0564	0.0415 (0.0123)	0.0007
TAP	0.0610 (0.0065)	0.0000	0.0058 (0.0088)	0.5086	-0.0080 (0.0068)	0.2397	0.0050 (0.0098)	0.6139
THA	0.0602 (0.0058)	0.0000	0.0050 (0.0084)	0.5564	-0.0066 (0.0062)	0.2812	0.0109 (0.0094)	0.2439
TTO	0.0645 (0.0073)	0.0000	0.0298 (0.0113)	0.0086	-0.0177 (0.0069)	0.0101	0.0416 (0.0107)	0.0001
TUN	0.0251 (0.0068)	0.0002	0.0134 (0.0112)	0.2311	-0.0438 (0.0060)	2.96e-13	0.0211 (0.0098)	0.0316
TUR	0.0508 (0.0066)	1.11e-14	0.0098 (0.0097)	0.3130	-0.0334 (0.0066)	3.31e-07	0.0242 (0.0101)	0.0163
URY	0.0462 (0.0062)	8.08e-14	0.0205 (0.0105)	0.0502	-0.0420 (0.0066)	1.99e-10	0.0273 (0.0106)	0.0103
USA	0.0244 (0.0070)	0.0005	0.0284 (0.0100)	0.0043	-0.0376 (0.0071)	1.14e-07	0.0259 (0.0106)	0.0147

Notes: Obtained by OLS estimations of Equation (3). Standard errors (in parentheses) clustered at the student level. The p-values are obtained from a two-sided t-test. Source data are provided as a Source Data file (Study 1).

Supplementary Table 3: Rotation design of the 20 PISA booklets. The PISA 2009.

Booklet	Cluster 1	Cluster 2	Cluster 3	Cluster 4	Standard booklet	Easy booklet
1	Math 1	Reading 1	Reading 3A	Math 3	X	
2	Reading 1	Science 1	Reading 4A	Reading 7	X	
3	Science 1	Reading 3A	Math 2	Science 3	X	
4	Reading 3A	Reading 4A	Science 2	Reading 2	X	
5	Reading 4A	Math 2	Reading 5	Math 1	X	
6	Reading 5	Reading 6	Reading 7	Reading 3A	X	
7	Reading 6	Math 3	Science 3	Reading 4A	X	
8	Reading 2	Math 1	Science 1	Reading 6	X	X
9	Math 2	Science 2	Reading 6	Reading 1	X	X
10	Science 2	Reading 5	Math 3	Science 1	X	X
11	Math 3	Reading 7	Reading 2	Math 2	X	X
12	Reading 7	Science 3	Math 1	Science 2	X	X
13	Science 3	Reading 2	Reading 1	Reading 5	X	X
14	Math 1	Reading 1	Reading 3B	Math 3		X
15	Reading 1	Science 1	Reading 4B	Reading 7		X
16	Science 1	Reading 3B	Math 2	Science 3		X
17	Reading 3B	Reading 4B	Science 2	Reading 2		X
18	Reading 4B	Math 2	Reading 5	Math 1		X
19	Reading 5	Reading 6	Reading 7	Reading 3B		X
20	Reading 6	Math 3	Science 3	Reading 4B		X

Source [3].

Supplementary Table 4: Randomization test. The PISA 2009.

	Gender	Mother highest schooling	Father highest schooling	Self born in country	Mother born in country	Father born in country	Language at home	Possessions desk	Possessions own room	How many books at home	Age of student
	(1)	(2)	(3)	(4)	(5)	(6)	(7)	(8)	(9)	(10)	(11)
Booklet=2	0.00794	0.00334	-0.0250	0.00291	0.00613	-0.00468	-0.00539	-0.00224	-0.00317	0.00143	0.00301
	(0.72)	(0.14)	(-1.05)	(0.63)	(0.82)	(-0.63)	(-0.67)	(-0.27)	(-0.38)	(0.04)	(0.47)
Booklet=3	0.00439	0.0172	0.0186	-0.00323	-0.00140	-0.00698	-0.000346	0.00149	-0.0111	-0.0142	-0.000698
	(0.40)	(0.73)	(0.75)	(-0.73)	(-0.19)	(-0.93)	(-0.04)	(0.18)	(-1.39)	(-0.45)	(-0.11)
Booklet=4	0.0117	0.000263	-0.00718	0.00160	0.00324	-0.00803	-0.0101	-0.00366	0.00676	0.0162	0.00872
	(1.07)	(0.01)	(-0.30)	(0.35)	(0.44)	(-1.09)	(-1.25)	(-0.45)	(0.82)	(0.53)	(1.35)
Booklet=5	0.0114	0.00391	-0.00166	-0.000857	0.000863	-0.00269	-0.00484	-0.00651	-0.00344	-0.0165	0.00185
	(1.03)	(0.17)	(-0.07)	(-0.16)	(0.11)	(-0.34)	(-0.58)	(-0.81)	(-0.42)	(-0.52)	(0.29)
Booklet=6	0.0217**	-0.0101	0.000509	-0.000475	0.00190	-0.000299	0.00311	-0.00292	-0.0180**	0.0122	0.00410
	(1.98)	(-0.44)	(0.02)	(-0.10)	(0.26)	(-0.04)	(0.38)	(-0.36)	(-2.27)	(0.39)	(0.64)
Booklet=7	-0.00131	0.0199	0.00347	0.0000170	0.00429	0.00278	0.00220	0.00548	-0.00509	-0.0189	-0.000367
	(-0.12)	(0.84)	(0.14)	(0.00)	(0.57)	(0.36)	(0.26)	(0.65)	(-0.63)	(-0.60)	(-0.05)
Booklet=8	0.00208	-0.00763	-0.00756	0.00177	-0.00378	-0.00552	-0.000470	0.00390	-0.00960	-0.0312	0.000895
	(0.21)	(-0.35)	(-0.34)	(0.43)	(-0.58)	(-0.83)	(-0.06)	(0.51)	(-1.28)	(-1.10)	(0.15)
Booklet=9	0.00432	-0.0159	0.00497	0.00103	-0.00364	-0.00882	-0.00105	0.00578	-0.00818	-0.0119	0.00527
	(0.43)	(-0.75)	(0.22)	(0.25)	(-0.56)	(-1.34)	(-0.14)	(0.74)	(-1.09)	(-0.42)	(0.88)
Booklet=10	-0.00596	-0.000868	0.00947	-0.000346	-0.00139	-0.00567	0.00107	-0.00366	-0.00347	0.0296	0.00404
	(-0.60)	(-0.04)	(0.42)	(-0.09)	(-0.21)	(-0.85)	(0.14)	(-0.50)	(-0.46)	(1.04)	(0.69)
Booklet=11	0.00889	-0.00605	0.00440	0.000531	-0.00311	-0.00455	-0.00606	0.00546	-0.00311	-0.0166	0.00177
	(0.89)	(-0.29)	(0.20)	(0.13)	(-0.48)	(-0.68)	(-0.82)	(0.72)	(-0.42)	(-0.58)	(0.30)
Booklet=12	0.00553	0.0135	-0.0144	0.00339	0.00105	-0.000688	-0.00439	-0.00778	-0.00332	-0.00415	0.00403
	(0.56)	(0.63)	(-0.64)	(0.83)	(0.16)	(-0.10)	(-0.59)	(-1.06)	(-0.44)	(-0.15)	(0.70)
Booklet=13	-0.00356	0.00992	0.000589	0.00158	-0.00142	-0.00236	0.000164	-0.000736	-0.00618	-0.0134	-0.00286
	(-0.36)	(0.46)	(0.03)	(0.39)	(-0.22)	(-0.35)	(0.02)	(-0.10)	(-0.82)	(-0.48)	(-0.49)
Booklet=14	-0.000470	-0.0186	-0.00923	0.00275	0.000141	-0.00382	0.00466	-0.00288	-0.0221**	-0.0260	0.00442
	(-0.04)	(-0.60)	(-0.29)	(0.69)	(0.02)	(-0.60)	(0.61)	(-0.28)	(-2.17)	(-0.84)	(0.63)
Booklet=15	-0.00152	-0.0244	-0.0345	-0.000293	-0.00381	-0.00464	0.000911	-0.00628	-0.00794	-0.0278	0.00395
	(-0.13)	(-0.79)	(-1.09)	(-0.08)	(-0.63)	(-0.74)	(0.12)	(-0.61)	(-0.77)	(-0.91)	(0.56)
Booklet=16	0.0100	-0.0191	-0.0488	0.00386	-0.00451	-0.00275	0.000977	0.00637	-0.00798	-0.0202	0.00402
	(0.84)	(-0.62)	(-1.55)	(0.92)	(-0.75)	(-0.43)	(0.13)	(0.63)	(-0.78)	(-0.66)	(0.58)
Booklet=17	-0.000417	0.0183	-0.00197	0.000248	-0.00377	-0.00455	0.000704	0.00921	-0.0104	-0.0320	0.0000957
	(-0.03)	(0.60)	(-0.06)	(0.06)	(-0.62)	(-0.72)	(0.09)	(0.90)	(-1.02)	(-1.06)	(0.01)
Booklet=18	0.00400	-0.0352	-0.0318	0.00196	0.00197	-0.000900	0.00388	0.00915	-0.0129	0.0303	0.000963
	(0.33)	(-1.16)	(-1.02)	(0.49)	(0.30)	(-0.14)	(0.49)	(0.90)	(-1.26)	(0.95)	(0.14)
Booklet=19	-0.00614	-0.0413	-0.0261	0.00291	-0.00258	-0.00562	0.00205	-0.0100	-0.0111	0.0140	-0.00498
	(-0.51)	(-1.33)	(-0.82)	(0.72)	(-0.43)	(-0.91)	(0.27)	(-1.00)	(-1.10)	(0.45)	(-0.72)
Booklet=20	0.00597	-0.0123	-0.00122	0.000691	-0.000498	-0.00226	-0.000666	-0.00227	0.00433	0.0218	-0.000613
	(0.50)	(-0.40)	(-0.04)	(0.18)	(-0.08)	(-0.35)	(-0.09)	(-0.23)	(0.41)	(0.70)	(-0.09)
Constant	1.511***	2.114***	2.010***	1.013***	1.010***	1.012***	1.010***	1.074***	1.323***	2.178***	15.77***
	(121.81)	(72.09)	(68.51)	(245.08)	(169.31)	(167.36)	(149.79)	(131.15)	(123.59)	(66.12)	(2178.87)
Observations	514865	486133	473178	506007	502761	499261	495177	504103	505341	504108	514867
F-value	0.82	0.64	0.62	0.47	0.56	0.48	0.58	0.86	1.10	1.25	0.55
p-value	0.689	0.879	0.893	0.976	0.936	0.970	0.925	0.632	0.342	0.208	0.941
Adjusted R^2	0.002	0.275	0.215	0.041	0.125	0.125	0.296	0.108	0.113	0.151	0.041

Notes: t statistics in parentheses
* $p < 0.10$, ** $p < 0.05$, *** $p < 0.01$
Regressions of background characteristics upon separate dummies for every booklet and country. We directly use the coding of the answer categories from the PISA (e.g. in column (1) we have an outcome variable where 1=male and 2=female), this only affects the interpretation of the constant. The columns F-value and p-value refer to F-tests for the joint significance of the booklet dummies. The PISA 2009 and PISA weights are used. Source data are provided as a Source Data file (Study 1).

Supplementary Table 5: When in the test do girls close the gender gap in math and science? Based upon estimates from the PISA 2009.

Country	Gap at beginning of test	After how long do boys and girls perform equal?
India (Himachal Pradesh)	-5.64^{++}	92 %
Malta	-3.95	72 %
Slovenia	-6.83^{++}	139 %
Jordan	-4.21^{++}	90 %
Serbia	-5.86^{++}	125 %
Ireland	-3.87^{++}	93 %
Trinidad and Tobago	-1.77^{++}	43 %
Sweden	-1.55	37 %
Montenegro	-3.77^{++}	96 %
Luxembourg	-5.41^{++}	139 %
Australia	-3.48^{++}	89 %
Norway	-2.01^{++}	52 %
Czech Republic	-5.54^{++}	156 %
New Zealand	-2.07^{++}	62 %
Latvia	-2.02^{++}	61 %
Brazil	-3.68^{++}	112 %
United Kingdom	-4.64^{++}	143 %
Denmark	-4.31^{++}	140 %
Austria	-8.41^{++}	274 %
China (Shanghai)	-3.06^{++}	103 %
Canada	-3.12^{++}	106 %
Liechtenstein	-8.33^{+}	297 %
Azerbaijan	-1.73^{++}	63 %
Indonesia	-2.07^{++}	75 %
Uruguay	-4.20^{++}	154 %
Hungary	-6.28^{++}	235 %
Mexico	-4.99^{++}	187 %
Italy	-6.69^{++}	253 %
Germany	-6.12^{++}	232 %
United States	-3.76^{++}	145 %
Portugal	-3.50^{++}	137 %
Colombia	-5.68^{++}	230 %
Turkey	-3.34^{++}	138 %
Greece	-4.04^{++}	172 %
Lithuania	-0.55	24 %
Qatar	-1.59	71 %
Korea	-2.55^{++}	114 %
United Arab Emirates	-1.04	48 %
Tunisia	-4.38^{++}	208 %
Switzerland	-4.82^{++}	238 %
Romania	-6.88^{++}	342 %
Mauritius	-3.53^{+}	190 %
Chile	-4.96^{+}	267 %
Spain	-3.60^{++}	195 %
Israel	-3.62^{+}	205 %
Poland	-1.52^{+}	86 %
Panama	-4.46^{+}	257 %
Slovak Republic	-3.37^{+}	195 %
Netherlands	-4.41^{+}	261 %
Costa Rica	-5.35^{+}	326 %
Singapore	-2.15^{+}	135 %
Belgium	-5.97^{+}	378 %
Iceland	-1.31	84 %
Japan	-2.96^{+}	204 %
Malaysia	-1.28	92 %
Thailand	-.066	61 %
Croatia	-5.15^{+}	576 %
France	-5.34^{+}	626 %
Russian Federation	-0.92	115 %
Bulgaria	-1.77^{+}	221 %
Peru	-3.88^{+}	578 %
China (Hong Kong)	-4.15^{+}	622 %
India (Tamil Nadu)	-1.46^{+}	239 %
Taiwan	-0.80	161 %

Notes: $^{+}$ indicates a significant gender difference at the start of the test and $^{++}$ indicates both a significant gender difference at the start of and during the test (at the 5% level). The gender gap at the beginning of the test can be interpreted as the percentage point difference to answer the first question correct (as measured by Equation (3) and subsequently multiplied by 100). The table includes countries where boys score better at the beginning of the test and girls perform better during the test in math and science. Source data are provided as a Source Data file (Study 1).

Supplementary Table 6: Overview of all the PISA items that are used by the PISA to construct the validated measures of students' noncognitive skills used in Study 1. Final column reports the gender differences (female-male) in the noncognitive skills.

Question	Noncognitive skill	Gender diff.
**PISA 2006**		
How much interest do you have in learning about the following:<br>- Topics in physics [4. High interest - 1. No interest]<br>- Topics in chemistry [4. High interest - 1. No interest]<br>- The biology of plants [4. High interest - 1. No interest]<br>- Human biology [4. High interest - 1. No interest]<br>- Topics in astronomy [4. High interest - 1. No interest]<br>- Topics in geology [4. High interest - 1. No interest]<br>- Ways scientists design experiments [4. High interest - 1. No interest]<br>- What is required for scientific explanations [4. High interest - 1. No interest]	Interest in science	0.000
Making an effort in science courses is worth it because this will help me in the work I want to do later on [4. Strongly agree - 1. Strongly disagree]<br>What I learned in my science courses is important for me because I need this for what I want to do later on [4. Strongly agree - 1. Strongly disagree]<br>I study science courses because I know it is useful for me [4. Strongly agree - 1. Strongly disagree]<br>Studying science courses is worthwhile for me because what I learn will improve my career prospects [4. Strongly agree - 1. Strongly disagree]<br>I will learn many things in my science courses that will help me get a job [4. Strongly agree - 1. Strongly disagree]	Instrumental motivation science	-0.016^{***}
I would like to work in a career involving science [4. Strongly agree - 1. Strongly disagree]<br>I would like to study science after secondary school [4. Strongly agree - 1. Strongly disagree]<br>I would like to spend my life doing advanced science [4. Strongly agree - 1. Strongly disagree]<br>I would like to work on science projects as an adult [4. Strongly agree - 1. Strongly disagree]	Career in science	-0.090^{***}
**PISA 2009**		
I read only if have to [4. Strongly disagree - 1. Strongly agree]<br>Reading is one of my favorite hobbies [1. Strongly disagree - 4. Strongly agree]<br>I like talking about books with other people [1. Strongly disagree - 4. Strongly agree]<br>I find it hard to finish books [4. Strongly disagree - 1. Strongly agree]<br>I feel happy if I receive a book as present [1. Strongly disagree - 4. Strongly agree]<br>For me, reading is a waste of time [4. Strongly disagree - 1. Strongly agree]<br>I enjoy going to a bookstore or a library [1. Strongly disagree - 4. Strongly agree]<br>I read only to get information that I need [4. Strongly disagree - 1. Strongly agree]<br>I cannot sit still and read for more than a few minutes [4. Strongly disagree - 1. Strongly agree]<br>I like to express my opinions about books I have read [1. Strongly disagree - 4. Strongly agree]<br>I like to exchange books with my friends [1. Strongly disagree - 4. Strongly agree]	Interest in reading	0.573^{***}
School has done little to prepare me for adult life [4. Strongly disagree - 1. Strongly agree]<br>School has been a waste of time [4. Strongly disagree - 1. Strongly agree]<br>*"I think the course material in this class is useful for me to learn"*<br>School helped give me confidence to make decisions [1. Strongly disagree - 4. Strongly agree]<br>School has taught me things which could be useful in a job [1. Strongly disagree - 4. Strongly agree]	Attitude towards school<br><br>*MSQL (motivation), [31]*	0.112^{***}

Overview of all the PISA measures (continued)

Question	Noncognitive skill	Gender diff.
**PISA 2012**		
I enjoy reading about mathematics [4. Strongly agree - 1. Strongly disagree]<br>I look forward to my mathematics lessons [4. Strongly agree - 1. Strongly disagree]<br>I do mathematics because I enjoy it [4. Strongly agree - 1. Strongly disagree]<br>I am interested in things I learn in mathematics [4. Strongly agree - 1. Strongly disagree]	Interest in math	-0.179^{***}
Making an effort in mathematics is worth it because this will help me in the work I want to do later on [4. Strongly agree - 1. Strongly disagree]<br>Learning mathematics is worthwhile for me because what I learn will improve my career prospects [4. Strongly agree - 1. Strongly disagree]<br>Mathematics is an important subject for me because I need it for what I want to study later on [4. Strongly agree - 1. Strongly disagree]<br>I will learn many things in mathematics that will help me get a job [4. Strongly agree - 1. Strongly disagree]	Instrumental motivation math	-0.148^{***}
School has done little to prepare me for adult life when I leave school [1. Strongly agree - 4. Strongly disagree]<br>School has been a waste of time [1. Strongly agree - 4. Strongly disagree	Attitude towards school	0.147^{***}
"I think the course material in this class is useful for me to learn"	*MSQL (motivation), [31]*	
School has helped give me confidence to make decisions [4. Strongly agree - 1. Strongly disagree]<br>School has taught me things which could be useful in a job [4. Strongly agree - 1. Strongly disagree]		
Trying hard at school will help me get a good job [4. Strongly agree - 1. Strongly disagree]<br>Trying hard at school will help me get into a good college [4. Strongly agree - 1. Strongly disagree]<br>I enjoy receiving good grades [4. Strongly agree - 1. Strongly disagree]	Attitude towards learning	0.155^{***}
"Getting a good grade in this class is the most satisfying thing for me right now"	*MSQL (motivation), [31]*	
Trying hard at school is important [4. Strongly agree - 1. Strongly disagree]		
Using a train timetable to work out how long it would take to get from one place to another [4. Very confident - 1. Not at all confident]<br>Calculating how much cheaper a TV would be after a 30% discount [4. Very confident - 1. Not at all confident]<br>Calculating how many square meters of tiles you need to cover a floor [4. Very confident - 1. Not at all confident]<br>Understanding graphs presented in newspapers [4. Very confident - 1. Not at all confident]<br>Solving an equation like $3x + 5 = 17$ [4. Very confident - 1. Not at all confident]<br>Finding the actual distance between two places on a map with a 1:10,000 scale [4. Very confident - 1. Not at all confident]<br>Solving an equation like $2(x + 3) = (x + 3)(x - 3)$ [4. Very confident - 1. Not at all confident]<br>Calculating the petrol consumption rate of a car [4. Very confident - 1. Not at all confident]	Self-efficacy in math	-0.276^{***}
I am just not good in mathematics [1. Strongly agree - 4. Strongly disagree]<br>I get good grades in mathematics [4. Strongly agree - 1. Strongly disagree]<br>I learn mathematics quickly [4. Strongly agree - 1. Strongly disagree]<br>I have always believed that mathematics is one of my best subjects [4. Strongly agree - 1. Strongly disagree]<br>In my mathematics class, I understand even the most difficult work [4. Strongly agree - 1. Strongly disagree]	Self-concept in math	-0.307^{***}

Overview of all the PISA measures (continued)

Question	Noncognitive skill	Gender diff.
I did an internship [2. Yes - 1. No never]<br>I did a work visit [2. Yes - 1. No never]<br>I visited a job fair [2. Yes - 1. No never]<br>I spoke to a career advisor at my school [2. Yes - 1. No never]<br>I spoke to a career advisor outside of my school [2. Yes - 1. No never]<br>I completed a questionnaire to find out about my interests and abilities [2. Yes - 1. No never]<br>I researched the internet for information about careers [2. Yes - 1. No never]<br>I went on an organised tour in a higher-education institution [2. Yes - 1. No never]<br>I researched the internet for a higher-education institution [2. Yes - 1. No never]	Career oriented	0.046^{***}
When confronted with a problem, I give up easily [1. Very much like me - 5. Not at all like me]	Conscientiousness	-0.032^{***}
"Setbacks do not discourage me"	*Gritt, [32]*	
I put off difficult problems. [1. Very much like me - 5. Not at all like me]<br>I remain interested in the tasks that I start [5. Very much like me - 1. Not at all like me]<br>I continue working on tasks until everything is perfect [5. Very much like me - 1. Not at all like me]		
"Perseveres until the task is finished"	*Conscientiousness, [6]*	
When confronted with a problem, I do more than what is expected of me [5. Very much like me - 1. Not at all like me]		
I can handle a lot of information [5. Very much like me - 1. Not at all like me]<br>I am quick to understand things [5. Very much like me - 1. Not at all like me]<br>I seek explanation for things [5. Very much like me - 1. Not at all like me]<br>I can easily link facts together [5. Very much like me - 1. Not at all like me]<br>I like to solve problems [5. Very much like me - 1. Not at all like me]	Openness (to problem solving)	-0.201^{***}
"Is ingenious, a deep thinker"	*Openness, [6]*	
I often worry that it will be difficult for me in mathematics classes [4. Strongly agree - 1. Strongly disagree]<br>I get very tense when I have to do mathematics homework [4. Strongly agree - 1. Strongly disagree]<br>I get very nervous doing mathematics problems [4. Strongly agree - 1. Strongly disagree]	Neuroticism (math)	0.232^{***}
"Gets nervous easily"	*Neuroticism, [6]*	
I feel helpless when doing mathematics problem [4 Strongly agree - 1. Strongly disagree]<br>I worry that I will get poor grades in mathematics [4. Strongly agree - 1. Strongly disagree]		
Imagine you have done bad on a recent mathematics quiz. What could explain this?<br>- I am not very good at solving mathematics problems [4. Very likely - 1. Not at all likely]<br>- My teacher did not explain the concepts well this week [4. Very likely - 1. Not at all likely]<br>- This week I made bad guesses on the quiz [4. Very likely - 1. Not at all likely]<br>- Sometimes the course material is too hard [4. Very likely - 1. Not at all likely]<br>- The teacher did not get students interested in the material [4. Very likely - 1. Not at all likely]<br>- Sometimes I am just unlucky [4. Very likely - 1. Not at all likely]	Locus of control (math)	0.078^{***}
"There really is no such thing as luck"	*Locus of control, [33]*	

Notes: The table displays the PISA questions that are used to construct the validated PISA measures used in Study 1. The measured noncognitive skill is displayed next to the first question in the list of all the questions that are used to measure that particular skill. More details on how the PISA constructs these measures can be found in the main text. For some individual questions the table shows similar questions of scales that have been validated by other research in *italics*.
The gender difference reflects the female dummy of a regression where the noncognitive skill is explained by a female dummy and country fixed effects. The regressions are estimated on the student level, where the sample includes all participating countries. The noncognitive skills are standardized and standard errors are clustered on the student level. * $p < 0.10$, ** $p < 0.05$, *** $p < 0.01$. Source: [2, 3, 4].

Supplementary Table 7: Overview of all the individual PISA background questions used in the analysis of student noncognitive skills in Study 1. Final column reports the gender differences (female-male) in the noncognitive skills.

Question	Noncognitive skill	Gender diff.
**PISA 2009**		
When I study, I try to relate new information to prior knowledge acquired in other subjects [1. Almost never - 4. Almost always]	Openness‡	0.012***
"Likes to reflect, play with ideas"	*Openness, [6]*	
I learn about things that are not course-related, such as sports, hobbies, people or music [1. Never - 5. Several times a week]	Openness‡	−0.021***
"Is sophisticated in art, music, or literature"	*Openness, [6]*	
Participate in online forums, virtual communities or spaces [1. Never - 4. Almost every day]	Openness‡	−0.040***
I get along well with most of my teachers [1. Strongly disagree - 4. Strongly agree]	Agreeableness	0.156***
"Starts quarrels with others"	*Agreeableness, [6]*	
**PISA 2012**		
I make friends easily at school [4. Strongly agree - 1. Strongly disagree]	Extraversion	−0.076***
"Is outgoing, sociable"	*Extraversion, [6]*	
If I put in enough effort, I can succeed in school [4. Strongly agree - 1. Strongly disagree]	Locus of control‡	0.078***
It is completely my choice whether or not I do well at school [4. Strongly agree - 1. Strongly disagree]	Locus of control‡	0.005*
"There is a direct connection between how hard I study and the grades I get"	*Locus of control, [33]*	
Family demands or other problems prevent me from putting a lot of time into my school work [1. Strongly agree - 4. Strongly disagree]	Locus of control‡	0.046***
If I had different teachers, I would try harder at school [1. Strongly agree - 4. Strongly disagree]	Locus of control‡	0.112***
If I wanted to, I could perform well at school [4. Strongly agree - 1. Strongly disagree]	Locus of control‡	−0.011***
I perform poorly at school whether or not I study for my exams [1. Strongly agree - 4. Strongly disagree]	Locus of control‡	0.148***

Notes: The table displays the individual items used in Study 1, the related noncognitive skills, and for some items it shows similar questions of scales that have been validated by previous research in *italics*.
For individual items that measure the same noncognitive skill within a PISA wave, we have also constructed the first principal component to avoid reliance on one individual item. These items are indicated with ‡.
The gender difference reflects the female dummy of a regression where the item is explained by a female dummy and country fixed effects. The regressions are estimated on the student level, where the sample includes all participating countries. The items are standardized and standard errors are clustered on the student level. * $p < 0.10$, ** $p < 0.05$, *** $p < 0.01$. Source: [3, 4].

Supplementary Table 8: Regression of the gender difference in sustaining performance during test on the gender difference in dynamic inputs during test.

	(1)	(2)	(3)	(4)	(5)	(6)
Gender difference in time during test	0.0843*** (4.44)	0.109*** (3.28)			0.0666*** (2.88)	0.0892*** (2.81)
Gender difference in actions during test			0.00174** (2.62)	0.00398*** (3.38)	0.000986 (1.30)	0.00282** (2.12)
Gender difference in time at the start		0.0134 (0.31)				0.0251 (0.67)
Gender difference in actions at the start				0.000162 (0.44)		-0.0000534 (-0.17)
Its interaction for time		-0.258 (-0.57)				-0.176 (-0.47)
Its interaction for actions				-0.000182 (-1.52)		-0.000163 (-1.31)
Constant	0.0167*** (17.29)	0.0153*** (5.54)	0.0194*** (14.36)	0.0192*** (8.28)	0.0182*** (12.87)	0.0170*** (5.48)
N	58	58	58	58	58	58
Adj. R^2	0.203	0.183	0.132	0.150	0.227	0.205

Notes: t statistics in parentheses, heteroskedasticity robust standard errors
* $p < 0.10$, ** $p < 0.05$, *** $p < 0.01$
The gender difference in ability to sustain performance is β_3 of Equation (2). The equations estimated are as follows: $\widehat{\beta_{3c}} = \alpha_0 + \alpha_1 \text{gdd}_c + \alpha_2 \text{gds}_c + \alpha_3 \text{gdd}_c \text{gds}_c + \epsilon_c$, where c is a subscript for country c and gdd and gds denote gender differences in inputs during the test and at the start of the test, respectively. Source data are provided as a Source Data file (Study 1).

Supplementary Table 9: Relationship between the gender gap in math and the length of a test.

	Whole sample	Exclude tests with noq≤ 10	Exclude tests with noq≤ 40	Whole sample	Recalculated gender gap	Weighted regression	Exclude five extreme long tests	Exclude tests with time≤ 5	Exclude tests with time≤ 20
	(1)	(2)	(3)	(4)	(5)	(6)	(7)	(8)	(9)
Number of questions	-0.00159**	-0.00347***	-0.00339**						
	(-2.06)	(-3.34)	(-2.09)						
Maximum time allowed				-0.000761	-0.000719	-0.000941	-0.00214***	-0.00244***	-0.00281***
				(-1.05)	(-0.99)	(-1.23)	(-2.73)	(-2.77)	(-3.37)
Constant	0.200***	0.337***	0.361***	0.180***	0.174***	0.195***	0.228***	0.249***	0.282***
	(4.59)	(5.70)	(3.02)	(3.54)	(3.38)	(3.87)	(4.27)	(4.05)	(4.59)
N	203	169	74	175	175	175	170	157	109
Adj. R^2	0.012	0.069	0.080	0.000	0.000	0.004	0.026	0.033	0.057

Notes: t statistics in parentheses, heteroskedasticity robust standard errors
* $p < 0.10$, ** $p < 0.05$, *** $p < 0.01$
The standardized math gender gap (mgp) is measured by subtracting the mean performance of girls from the mean performance of boys and dividing this by the pooled standard deviation. The equations estimated are as follows: $\text{mgp}_i = \delta_0 + \delta_1 \text{length}_i + w_i$, where i is a subscript for test i and length_i is either the number of questions (columns (1) to (3)) or the maximum time allowed to complete the test (columns (4) to (9)). Source data are provided as a Source Data file (Study 2).

Supplementary Table 10: Gender gap in math and number of questions on a test.

	Whole sample	Australia, Europe and Middle East	Asia	Tests with stakes
	(1)	(2)	(3)	(4)
Number of questions	-0.00159**	-0.00699***	0.00183	-0.00557*
	(-2.06)	(-3.48)	(0.91)	(-1.87)
Constant	0.200***	0.380***	0.0907	0.192
	(4.59)	(4.36)	(1.65)	(1.10)
N	203	45	20	17
Adj. R^2	0.012	0.303	0.005	0.151

Notes: t statistics in parentheses, heteroskedasticity robust standard errors
* $p < 0.10$, ** $p < 0.05$, *** $p < 0.01$
The standardized math gender gap (mgp) is measured by subtracting the mean performance of girls from the mean performance of boys and dividing this by the pooled standard deviation. The equations estimated are as follows: $\text{mgp}_i = \delta_0 + \delta_1 \text{noq}_i + w_i$, where i is a subscript for test i and noq_i denotes the number of questions on the test.
Column (4) includes the tests that are reported to have (at least some) stakes. This information is coded from the original articles in the meta-analysis of [22]. Source data are provided as a Source Data file (Study 2).

Supplementary Table 11: Gender differences in sustaining performance during the test while using probit. Testing the marginal effects, no distinction per topic. The PISA 2009.

Country	Gender diff. in marg. effects	Welsch t-test	Country	Gender diff. in marg. effects	Welsch t-test	Country	Gender diff. in marg. effects	Welsch t-test
ALB	0.0099	1.3291	HRV	0.0285	4.3271	NZL	0.0337	5.0439
ARE	0.0340	7.3498	HUN	0.0301	4.4991	PAN	0.0092	1.1907
ARG	0.0081	1.0089	IDN	0.0159	2.3504	PER	0.0023	0.3177
AUS	0.0385	9.7755	IRL	0.0473	6.2153	POL	0.0266	3.8571
AUT	0.0372	6.3322	ISL	0.0164	2.0786	PRT	0.0308	5.2138
AZE	0.0101	1.3851	ISR	0.0236	3.3301	QAT	0.0389	7.7825
BEL	0.0237	4.7554	ITA	0.0324	11.6104	QCN	0.0245	4.2256
BGR	0.0102	1.3467	JOR	0.0562	8.8016	QHP	0.0333	2.9674
BRA	0.0380	10.7772	JPN	0.0138	2.2962	QTN	0.0138	1.8550
CAN	0.0377	12.1427	KAZ	-0.0016	-0.2429	QVE	-0.0059	-0.6024
CHE	0.0306	7.0960	KGZ	0.0041	0.5591	ROU	0.0216	3.1391
CHL	0.0279	4.3418	KOR	0.0214	3.5306	RUS	0.0143	1.9865
COL	0.0210	3.5763	LIE	0.0327	1.1886	SGP	0.0101	1.6584
CRI	0.0180	2.5292	LTU	0.0269	3.9264	SRB	0.0290	4.4584
CZE	0.0338	5.5494	LUX	0.0245	3.3317	SVK	0.0192	2.6880
DEU	0.0271	4.1157	LVA	0.0357	5.0938	SVN	0.0512	8.5132
DNK	0.0340	5.5929	MAC	-0.0034	-0.5473	SWE	0.0442	6.1275
ESP	0.0224	7.3424	MDA	0.0043	0.5842	TAP	0.0071	1.1970
EST	0.0095	1.4582	MEX	0.0200	8.0478	THA	0.0103	1.7450
FIN	0.0267	4.5598	MLT	0.0586	6.6506	TTO	0.0370	4.7927
FRA	0.0266	3.5742	MNE	0.0279	4.0536	TUN	0.0211	2.8732
GBR	0.0312	7.5104	MUS	0.0192	2.8025	TUR	0.0194	2.8759
GEO	0.0044	0.5425	MYS	0.0147	2.0660	URY	0.0248	3.4295
GRC	0.0212	2.7607	NLD	0.0215	3.4182	USA	0.0300	4.7687

Notes: See Supplementary Note 3 and Supplementary Equation (1) for the exact procedure of testing the statistical significance of the marginal effects. Source data are provided as a Source Data file (Study 1).

Supplementary Table 12: Nonlinear Wald test on gender differences in sustaining performance (sust. perf.), for the whole test (all topics) and per topic (reading and math-science). The PISA 2009.

Country	Gender diff. in sust. perf. (all topics)	p-value (all topics)	Gender diff. in sust. perf. is statistically significant (5%)	Gender diff. in sust. perf. (reading)	p-value (reading)	Gender diff. in sust. perf. is statistically significant (5%)	Gender diff. in sust. perf. (math-science)	p-value (math-science)	Gender diff. in sust. perf. is statistically significant (5%)
ALB	0.054	0.001	Yes	0.078	0.001	Yes	-0.001	0.963	
ARE	0.054	3.70e-14	Yes	0.067	2.85e-11	Yes	0.033	0.003	Yes
ARG	0.018	0.268		0.049	0.022	Yes	-0.034	0.161	
AUS	0.042	7.23e-24	Yes	0.039	1.58e-10	Yes	0.040	5.23e-08	Yes
AUT	0.088	5.39e-07	Yes	0.145	9.30e-06	Yes	0.056	0.034	Yes
AZE	0.025	0.045	Yes	0.020	0.424		0.048	0.009	Yes
BEL	0.027	1.09e-06	Yes	0.032	0.000	Yes	0.015	0.102	
BGR	0.034	0.053		0.043	0.055		0.011	0.623	
BRA	0.048	8.12e-27	Yes	0.060	4.96e-20	Yes	0.037	1.16e-09	Yes
CAN	0.045	8.38e-32	Yes	0.050	7.08e-21	Yes	0.032	1.96e-07	Yes
CHE	0.042	6.76e-14	Yes	0.055	6.95e-11	Yes	0.022	0.017	Yes
CHL	0.038	0.000	Yes	0.043	0.000	Yes	0.020	0.149	
COL	0.040	0.004	Yes	0.029	0.022	Yes	0.036	0.082	
CRI	0.025	0.024	Yes	0.021	0.082		0.018	0.276	
CZE	0.034	5.00e-10	Yes	0.034	0.000	Yes	0.032	0.001	Yes
DEU	0.027	0.000	Yes	0.027	0.002	Yes	0.023	0.018	Yes
DNK	0.040	2.10e-08	Yes	0.043	0.000	Yes	0.033	0.007	Yes
ESP	0.028	2.19e-14	Yes	0.032	7.58e-10	Yes	0.019	0.002	Yes
EST	0.017	0.048	Yes	0.025	0.025	Yes	-0.003	0.845	
FIN	0.037	8.95e-08	Yes	0.049	2.05e-06	Yes	0.016	0.185	
FRA	0.030	0.000	Yes	0.042	0.000	Yes	0.005	0.716	
GBR	0.044	3.69e-13	Yes	0.040	1.05e-06	Yes	0.042	0.000	Yes
GEO	0.066	0.010	Yes	0.077	0.011	Yes	0.043	0.083	
GRC	0.034	0.001	Yes	0.036	0.007	Yes	0.021	0.177	
HKG	0.018	0.043	Yes	0.024	0.038	Yes	0.006	0.683	
HRV	0.050	2.70e-06	Yes	0.065	1.61e-07	Yes	0.005	0.804	
HUN	0.037	0.000	Yes	0.038	0.001	Yes	0.030	0.035	Yes
IDN	0.035	0.003	Yes	0.021	0.160		0.049	0.005	Yes
IRL	0.064	2.27e-10	Yes	0.062	6.75e-06	Yes	0.054	0.002	Yes
ISL	0.023	0.012	Yes	0.023	0.102		0.018	0.265	
ISR	0.054	0.000	Yes	0.046	0.004	Yes	0.024	0.292	
ITA	0.034	2.53e-29	Yes	0.039	3.40e-21	Yes	0.022	1.93e-06	Yes
JOR	0.107	6.39e-14	Yes	0.092	8.33e-10	Yes	0.085	0.000	Yes
JPN	0.016	0.008	Yes	0.016	0.052		0.013	0.193	
KAZ	0.007	0.485		0.017	0.329		0.000	0.977	
KGZ	0.044	0.036	Yes	0.095	0.012	Yes	0.022	0.449	
KOR	0.033	0.000	Yes	0.027	0.017	Yes	0.030	0.045	Yes

Nonlinear Wald test (continued)

Country	Gender diff. in sust. perf. (all topics)	p-value (all topics)	Gender diff. in sust. perf. is statistically significant (5%)	Gender diff. in sust. perf. (reading)	p-value (reading)	Gender diff. in sust. perf. is statistically significant (5%)	Gender diff. in sust. perf. (math-science)	p-value (math-science)	Gender diff. in sust. perf. is statistically significant (5%)
LIE	0.031	0.225		0.034	0.354		0.023	0.582	
LTU	0.052	1.00e-06	Yes	0.049	0.000	Yes	0.037	0.032	Yes
LUX	0.038	0.000	Yes	0.029	0.073		0.051	0.004	Yes
LVA	0.052	3.73e-09	Yes	0.065	3.14e-06	Yes	0.043	0.004	Yes
MAC	-0.003	0.717		0.003	0.714		-0.014	0.267	
MDA	0.043	0.027	Yes	0.194	0.017	Yes	0.006	0.831	
MEX	0.024	8.50e-14	Yes	0.015	0.000	Yes	0.029	3.57e-09	Yes
MLT	0.066	2.48e-09	Yes	0.063	0.000	Yes	0.065	0.000	Yes
MNE	0.072	3.99e-07	Yes	0.068	0.001	Yes	0.079	0.001	Yes
MUS	0.020	0.012	Yes	0.017	0.108		0.020	0.122	
MYS	0.024	0.017	Yes	0.038	0.047	Yes	0.020	0.183	
NLD	0.021	0.000	Yes	0.024	0.004	Yes	0.014	0.143	
NOR	0.062	1.17e-12	Yes	0.069	1.43e-08	Yes	0.047	0.001	Yes
NZL	0.041	1.00e-07	Yes	0.037	0.001	Yes	0.038	0.007	Yes
PAN	0.008	0.483		-0.002	0.894		0.020	0.197	
PER	-0.012	0.568		-0.006	0.784		-0.015	0.623	
POL	0.040	2.78e-06	Yes	0.049	0.000	Yes	0.022	0.148	
PRT	0.046	1.29e-08	Yes	0.048	4.70e-06	Yes	0.032	0.018	Yes
QAT	0.062	3.77e-14	Yes	0.114	2.96e-13	Yes	0.035	0.003	Yes
QCN	0.039	3.74e-06	Yes	0.026	0.004	Yes	0.041	0.004	Yes
QHP	0.102	0.005	Yes	0.041	0.444		0.195	0.000	Yes
QTN	0.069	0.060		0.145	0.015	Yes	0.011	0.840	
QVE	-0.006	0.610		0.009	0.568		-0.027	0.136	
ROU	0.021	0.037	Yes	0.022	0.074		0.019	0.257	
RUS	0.021	0.005	Yes	0.030	0.005	Yes	0.008	0.515	
SGP	0.021	0.039	Yes	0.013	0.348		0.025	0.159	
SRB	0.106	8.54e-06	Yes	0.027	0.044	Yes	0.126	0.001	Yes
SVK	0.031	0.001	Yes	0.036	0.007	Yes	0.020	0.212	
SVN	0.061	4.86e-14	Yes	0.062	2.61e-09	Yes	0.054	6.10e-06	Yes
SWE	0.060	1.86e-10	Yes	0.066	6.89e-06	Yes	0.053	0.001	Yes
TAP	0.009	0.094		0.010	0.208		0.004	0.637	
THA	0.039	0.001	Yes	0.022	0.046	Yes	0.022	0.260	
TTO	0.231	9.66e-08	Yes	0.151	4.71e-07	Yes	0.165	0.000	Yes
TUN	0.028	0.022	Yes	0.023	0.071		0.026	0.134	
TUR	0.027	0.001	Yes	0.018	0.066		0.029	0.029	Yes
URY	0.033	0.000	Yes	0.038	0.003	Yes	0.029	0.031	Yes
USA	0.042	2.45e-06	Yes	0.041	0.001	Yes	0.032	0.030	Yes

Notes: See Supplementary Note 3 for the exact procedure of the nonlinear Wald test. The p-values are obtained from a nonlinear Wald test. Source data are provided as a Source Data file (Study 1).

Supplementary References

[1] OECD. *PISA 2015 Technical Report.* OECD Publishing, (2015).

[2] OECD. *PISA 2006 Technical Report.* OECD Publishing, (2009).

[3] OECD. *PISA 2009 Technical Report.* OECD Publishing, (2012).

[4] OECD. *PISA 2012 Technical Report.* OECD Publishing, (2014).

[5] Bong, M. and Skaalvik, E. M. Academic self-concept and self-efficacy: How different are they really? *Educational Psychology Review* **15**, 1–40 (2003).

[6] John, O. P. and Srivastava, S. The Big Five trait taxonomy: History, measurement, and theoretical perspectives. *Handbook of Personality: Theory and Research* Ch. 4, (Guilford Press, New York, 1999).

[7] Zamarro, G., Nichols, M., Duckworth, A., and D' Mello, S. Further validation of survey-effort measures of conscientiousness: Results from a sample of high school students. Preprint at: `https://ssrn.com/abstract=3265332` (2018).

[8] Zamarro, G., Cheng, A., Shakeel, M. D., and Hitt, C. Comparing and validating measures of non-cognitive traits: Performance task measures and self-reports from a nationally representative internet panel. *Journal of Behavioral and Experimental Economics* **72**, 51 – 60 (2018).

[9] Cheng, A., Zamarro, G., and Orriens, B. Personality as a predictor of unit nonresponse in an internet panel. *(in press). Sociological Methods & Research* (2018).

[10] Vodanovich, S. J., Wallace, J. C., and Kass, S. J. A confirmatory approach to the factor structure of the boredom proneness scale: Evidence for a two-factor short form. *Journal of Personality Assessment* **85**, 295–303 (2005).

[11] Vodanovich, S. J. and Kass, S. J. Age and gender differences in boredom proneness. *Journal of Social Behavior and Personality* **5**, 297–307 (1990).

[12] Zuckerman, M., Eysenck, S. B., and Eysenck, H. J. Sensation seeking in England and America: Cross-cultural, age, and sex comparisons. *Journal of Consulting and Clinical Psychology* **46**, 139–149 (1978).

[13] Eastwood, J. D., Frischen, A., Fenske, M. J., and Smilek, D. The unengaged mind: Defining boredom in terms of attention. *Perspectives on Psychological Science* **7**, 482–495 (2012).

[14] Kass, S. J., Beede, K. E., and Vodanovich, S. J. Self-report measures of distractibility as correlates of simulated driving performance. *Accident Analysis & Prevention* **42**, 874 – 880 (2010).

[15] Fisher, C. D. Boredom at work: A neglected concept. *Human Relations* **46**, 395–417 (1993).

[16] Malkovsky, E., Merrifield, C., Goldberg, Y., and Danckert, J. Exploring the relationship between boredom and sustained attention. *Experimental Brain Research* **221**, 59–67 (2012).

[17] Vodanovich, S. J. and Kass, S. J. A factor analytic study of the boredom proneness scale. *Journal of Personality Assessment* **55**, 115–123 (1990).

[18] Harris, M. B. Correlates and characteristics of boredom proneness and boredom. *Journal of Applied Social Psychology* **30**, 576–598 (2000).

[19] Buser, T. and Yuan, H. Do women give up competing more easily? Evidence from the lab and the Dutch Math Olympiad. Preprint at: `https://ssrn.com/abstract=2867346`, (2016).

[20] Norton, E. C., Wang, H., Ai, C., et al. Computing interaction effects and standard errors in logit and probit models. *Stata Journal* **4**, 154–167 (2004).

[21] Cameron, A. C. and Trivedi, P. K. *Microeconometrics: Methods and applications.* (Cambridge University Press, Cambridge (MA), 2005).

[22] Lindberg, S. M., Hyde, J. S., Petersen, J. L., and Linn, M. C. New trends in gender and mathematics performance: A meta-analysis. *Psychological Bulletin* **136**, 1123–1135 (2010).

[23] Croson, R. and Gneezy, U. Gender differences in preferences. *Journal of Economic Literature* **47**, 448–474 (2009).

[24] Gneezy, U., Niederle, M., Rustichini, A. Performance in competitive environments: Gender differences. *Quarterly Journal of Economics* **118**, 1049–1074 (2003).

[25] Gneezy, U. and Rustichini, A. Gender and competition at a young age. *American Economic Review* **94**, 377–381 (2004).

[26] Azmat, G., Calsamiglia, C., and Iriberri, N. Gender differences in response to big stakes. *Journal of the European Economic Association* **14**(6), 1372–1400 (2016).

[27] Iriberri, N. and Rey-Biel, P. Competitive pressure widens the gender gap in performance: Evidence from a two–stage competition in mathematics. *The Economic Journal* **129**, 1863–1893 (2019).

[28] Ors, E., Palomino, F., and Peyrache, E. Performance gender gap: Does competition matter? *Journal of Labor Economics* **31**, 443–499 (2013).

[29] Gneezy, U., List, J. A., Livingston, J. A., Sadoff, S., Qin, X., and Xu, Y. Measuring success in education: The role of effort on the test itself. Preprint at: `https://ssrn.com/abstract=3070028` (2017).

[30] Sjøberg, S. PISA and "real life challenges": Mission impossible? in *PISA according to PISA Ch.9* (LIT Verlag, Berlin, 2007).

[31] Duncan, T. G. and McKeachie, W. J. The making of the motivated strategies for learning questionnaire. *Educational Psychologist* **40**, 117–128 (2005).

[32] Duckworth, A. L., Peterson, C., Matthews, M. D., and Kelly, D. R. Grit: perseverance and passion for long-term goals. *Journal of Personality and Social Psychology* **92**, 1087–101 (2007).

[33] Rotter, J. B. Generalized expectancies for internal versus external control of reinforcement. *Psychological Monographs: General and Applied* **80**, (1966).
