# Supplementary material for: Females show more sustained performance during test-taking than males
Source: Nat Commun. 2019 Sep 3;10:3798. doi: 10.1038/s41467-019-11691-y (PMC6722057; doi:10.1038/s41467-019-11691-y)
Supplement: Supplementary file 3 — Reporting Summary [file 41467_2019_11691_MOESM3_ESM.pdf]

nature research

Corresponding author(s): Pau Balart

Last updated by author(s): Jul 2, 2019

Reporting Summary

Nature Research wishes to improve the reproducibility of the work that we publish. This form provides structure for consistency and transparency in reporting. For further information on Nature Research policies, see Authors & Referees and the Editorial Policy Checklist.

Statistics

For all statistical analyses, confirm that the following items are present in the figure legend, table legend, main text, or Methods section.

n/a	Confirmed	
☒	☐	The exact sample size (n) for each experimental group/condition, given as a discrete number and unit of measurement
☐	☒	A statement on whether measurements were taken from distinct samples or whether the same sample was measured repeatedly
☐	☒	The statistical test(s) used AND whether they are one- or two-sided<br>*Only common tests should be described solely by name; describe more complex techniques in the Methods section.*
☐	☒	A description of all covariates tested
☐	☒	A description of any assumptions or corrections, such as tests of normality and adjustment for multiple comparisons
☐	☒	A full description of the statistical parameters including central tendency (e.g. means) or other basic estimates (e.g. regression coefficient) AND variation (e.g. standard deviation) or associated estimates of uncertainty (e.g. confidence intervals)
☐	☒	For null hypothesis testing, the test statistic (e.g. F, t, r) with confidence intervals, effect sizes, degrees of freedom and P value noted<br>*Give P values as exact values whenever suitable.*
☒	☐	For Bayesian analysis, information on the choice of priors and Markov chain Monte Carlo settings
☒	☐	For hierarchical and complex designs, identification of the appropriate level for tests and full reporting of outcomes
☐	☒	Estimates of effect sizes (e.g. Cohen's d, Pearson's r), indicating how they were calculated

Our web collection on statistics for biologists contains articles on many of the points above.

Software and code

Policy information about availability of computer code

Data collection	We have used publicly available data to conduct our study. The PISA data used in Study 1 can be freely downloaded from the OECD website. Study 1 requires some processing of these data, which has been done with StataMP 14 software. This process is shortly described in the readme file of the Source Data file of Study 1. The Stata code to transform the publicly available PISA data into the long format provided in the Source Data file is available upon request from the corresponding author.
Data analysis	All the statistical analysis was conducted using StataMP 14 software. The Code File made available via the public repository hosted by OSF [http://doi.org/10.17605/OSF.IO/V5KQY] contains the Stata code that replicates all the results reported in the figures and tables of the article.

For manuscripts utilizing custom algorithms or software that are central to the research but not yet described in published literature, software must be made available to editors/reviewers. We strongly encourage code deposition in a community repository (e.g. GitHub). See the Nature Research guidelines for submitting code & software for further information.

Data

Policy information about availability of data

All manuscripts must include a data availability statement. This statement should provide the following information, where applicable:

- Accession codes, unique identifiers, or web links for publicly available datasets
- A list of figures that have associated raw data
- A description of any restrictions on data availability

The Source Data file and the Supplementary Databases are available in a public repository hosted by Open Science Framework (OSF). It can be downloaded via the following url: [http://doi.org/10.17605/OSF.IO/V5KQY].

The source data underlying Figs 1, 2, 3a-b, and 4a-b, Supplementary Figs 1, 2a-b, 3a-b, 4, 5, 6, 7, 8a-b, 9, 10, 11a-b, 12, 13, 14, 15, 16, 17a-b, 18, 19, 20, and Supplementary Tables 1, 2, 4, 5, 8, 11, and 12 are provided as a Source Data file (Study 1). The source data underlying Table 1 and Supplementary Table 9 and 10

are provided as a Source Data file (Study 2).

Field-specific reporting

Please select the one below that is the best fit for your research. If you are not sure, read the appropriate sections before making your selection.

☐ Life sciences ☒ Behavioural & social sciences ☐ Ecological, evolutionary & environmental sciences

For a reference copy of the document with all sections, see nature.com/documents/nr-reporting-summary-flat.pdf

Behavioural & social sciences study design

All studies must disclose on these points even when the disclosure is negative.

Study description

This article analyses gender differences in performance during a cognitive test. The data is quantitative. In study 1, we use the PISA data from 15-year-old students. In study 2, we use data on the math gender gap from multiple cognitive tests.

Research sample

Study 1: the research sample are 15-year-old students that are sampled by the PISA. Countries from all over the world participate with PISA as to evaluate student skills in math, science, and reading. With their two-stage sampling procedure (first schools, then students), PISA tries to create a representative sample. We describe this procedure in detail in the Methods section, where we also elaborate that this is not crucial for the interpretation of our results.

Study 2: the research sample are math tests, for which we have information about the gender gap and the number of questions. We work with the math tests that are present in the meta-analysis of Lindberg and co-authors.

Sampling strategy

Study 1: the PISA uses a two-stage stratified sample design. The first-stage sampling units consist of individual schools being sampled from a comprehensive national list of all PISA-eligible schools. The second-stage sampling units are the students. Once schools are selected, a complete list of all 15-year-old students in the school is prepared. If this list contains more than 35 students, 35 of them are randomly selected. Though depending somewhat upon the country, the PISA samples many students for each country, which means we have the statistical power to analyze gender differences.

Study 2: the research sample are math tests, for which we have information about the gender gap and the number of questions. We work with the math tests that are present in the meta-analysis of Lindberg and co-authors. Though our analysis is based upon roughly 200 tests, we have the statistical power to reject null effects.

For both studies we did not conduct a power analyses. We work with observational data.

Data collection

Study 1: the data collection was done by the PISA and subsequently made publicly available on the OECD website. The data collection has been carefully described in the publicly available technical reports of the PISA.

Study 2: we work with the math tests that are present in the meta-analysis of Lindberg and co-authors. Their meta-analysis involved the identification of possible studies that investigated the performance on math tests. By using computerized database searches, they generated a pool of potential articles. After careful selection, the final sample of studies included data from 441 math tests. For every test, the standardized gender gap was calculated and stored in a dataset. For every test in their dataset, we attempted to collect the following information from the original articles: the number of questions, the maximum time allowed to complete the test, and the stakes of the exam. If this information was not available in the original studies, we sent the authors an email asking for the information.

Timing

Study 1: data collection was done in 2015 for the PISA 2006, 2009, and 2012 and done in 2017 for the PISA 2015.

Study 2: Sara Lindberg kindly provided us with the original data on June 08, 2016.

Data exclusions

Study 1: if we observed the gender of the student in the PISA data, which is virtually true for every student, then he or she is included in the analysis. Hence, no data is excluded from the baseline analysis. When we include student measures for noncognitive skills in the model, we can only analyze the students that filled in the PISA background questionnaire (i.e., for which we have measures on noncognitive skills).

Study 2: for 243 out of the 441 tests included in the original dataset from Lindberg and co-authors (see above), we found evidence that they had to be completed within a certain time limit. Only these tests are of interest; without a limit of time, there is no reason that a test should measure sustained performance. Tests without a time limit are, for example, tests that are conducted at home or not during class time. For 203 of the 243 tests, we were able to collect the number of questions, and for 175 exams, we collected the maximum time allowed to complete the test. Sample attrition does not seem to be a problem for two reasons. First, when we compare the average size of the gender gap on tests with a time limit to the gender gap on tests without a time limit and for which we did not observe information about the time limit, we find that they are not significantly different. Second, for tests with a time limit, observing the number of questions does not correlate with the size of the gender gap.

Non-participation

Study 1: PISA samples 35 students randomly within each sampled school. Not all students, however, show up at the day of the test. PISA requires a response rate of 80% of the students within each school. Further details can be found in the technical reports of PISA. We then work with the data that the PISA makes publicly available.

Study 2: we work with the math tests that are present in the meta-analysis of Lindberg and co-authors. Their meta-analysis involved the

	identification of possible studies that investigated the performance on math tests. By using computerized database searches, they generated a pool of potential articles. After careful selection, the final sample of studies included data from 441 math tests.
Randomization	Study 1: clusters of questions vary in order between the PISA booklets and booklets are randomly handed out to students. As such, there is random variation in the position of a question among different students.<br><br>Study 2: we do not have exogenous variation in the length of the test. This means that Study 2 identifies correlations/associations. In the Discussion section of the paper we therefore write: "However, caution is needed for at least two reasons. First, Study 2 does not exploit exogenous variation in test length. This makes a causal interpretation of the results challenging. Second, (...)".

Reporting for specific materials, systems and methods

We require information from authors about some types of materials, experimental systems and methods used in many studies. Here, indicate whether each material, system or method listed is relevant to your study. If you are not sure if a list item applies to your research, read the appropriate section before selecting a response.

Materials & experimental systems

n/a	Involved in the study
☒	☐ Antibodies
☒	☐ Eukaryotic cell lines
☒	☐ Palaeontology
☒	☐ Animals and other organisms
☐	☒ Human research participants
☒	☐ Clinical data

Methods

n/a	Involved in the study
☒	☐ ChIP-seq
☒	☐ Flow cytometry
☒	☐ MRI-based neuroimaging

Human research participants

Policy information about studies involving human research participants

Population characteristics	See above
Recruitment	The recruitment is done by the PISA via a two-stage stratified sample design.
Ethics oversight	The OECD and the corresponding participating countries approve the study protocol.

Note that full information on the approval of the study protocol must also be provided in the manuscript.
